# Utilization of natural alleles for heat adaptability QTLs at the flowering stage in rice

**DOI:** 10.1186/s12870-023-04260-5

**Published:** 2023-05-16

**Authors:** Ying-Hua Pan, Lei Chen, Xiao-Yang Zhu, Jing-Cheng Li, Muhammad Abdul Rehman Rashid, Chao Chen, Dong-Jin Qing, Wei-Yong Zhou, Xing-Hai Yang, Li-Jun Gao, Yan Zhao, Guo-Fu Deng

**Affiliations:** 1 Rice Research Institute, Guangxi Academy of Agricultural Sciences/Guangxi Key Laboratory of Rice Genetics and Breeding, Nanning, 530007 China; 2 State Key Laboratory of Agrobiotechnology/Beijing Key Laboratory of Crop Genetic Improvement, College of Agronomy and Biotechnology, China Agricultural University, Beijing, 100193 China; 3 Department of Bioinformatics and Biotechnology, Government College University Faisalabad, Faisalabad, 38000 Pakistan; 4 State Key Laboratory of Crop Germplasm Innovation and Molecular Breeding/Life Science and Technology Center, China National Seed Group Co., LTD, Wuhan, 430206 China; 5 State Key Laboratory of Crop Biology, Shandong Key Laboratory of Crop Biology, College of Agronomy, Shandong Agricultural University, Tai’an, 271018 China

**Keywords:** GWAS, Heat stress, Quality, Gene pyramiding, Rice

## Abstract

**Background:**

Heat stress threatens rice yield and quality at flowering stage. In this study, average relative seed setting rate under heat stress (RHSR) and genotypes of 284 varieties were used for a genome-wide association study.

**Results:**

We identified eight and six QTLs distributed on chromosomes 1, 3, 4, 5, 7 and 12 in the full population and *indica*, respectively. *qHTT4.2* was detected in both the full population and *indica* as an overlapping QTL. RHSR was positively correlated with the accumulation of heat-tolerant superior alleles (SA), and *indica* accession contained at least two heat-tolerant SA with average RHSR greater than 43%, meeting the needs of stable production and heat-tolerant QTLs were offer yield basic for chalkiness degree, amylose content, gel consistency and gelatinization temperature. Chalkiness degree, amylose content, and gelatinization temperature under heat stress increased with accumulation of heat-tolerant SA. Gel consistency under heat stress decreased with polymerization of heat-tolerant SA. The study revealed *qHTT4.2* as a stable heat-tolerant QTL that can be used for breeding that was detected in the full population and *indica*. And the grain quality of *qHTT4.2-*haplotype1 (Hap1) with *chalk5*, *wx*, and *alk* was better than that of *qHTT4.2-*Hap1 with *CHALK5*, *WX*, and *ALK.* Twelve putative candidate genes were identified for *qHTT4.2* that enhance RHSR based on gene expression data and these genes were validated in two groups. Candidate genes *LOC_Os04g52830* and *LOC_Os04g52870* were induced by high temperature.

**Conclusions:**

Our findings identify strong heat-tolerant cultivars and heat-tolerant QTLs with great potential value to improve rice tolerance to heat stress, and suggest a strategy for the breeding of yield-balance-quality heat-tolerant crop varieties.

**Supplementary Information:**

The online version contains supplementary material available at 10.1186/s12870-023-04260-5.

## Background

With the growing world population and extreme weather occurrences, an estimated 702 to 828 million people around the world faced hunger in 2021 [1]. It is estimated that a 70% increase in food production is required to feed the expected population of 10 billion people in 2050 [2]. Each global temperature increases of 1 °C corresponds to a 3–8% reduction in the global mean yield of crops [3]. Rice (*Oryza sativa* L.) is a world staple crop that provides a source of energy for nearly half of the world population [4]. However, heat stress can severely affect the yield and quality of rice by reducing seed setting rate and influencing chalkiness degree, amylose content, gelatinization temperature, and gel consistency [5–8]. Exploring heat-tolerant QTLs/gene is a useful way to improve seed setting rate [9], but few heat-tolerant QTLs/genes involved in heat tolerance have been identified in rice, and the relationship between heat-tolerant QTLs and tolerance to high-temperature stress remains unclear.

Rice grows best within a temperature range of 20–30 °C during its reproductive stage. A previous study showed that degradation and disintegration of the tapetal cells impair the nutritional supply of microspores and reduce the spikelet fertility rate under high temperature stress [10]. Heat stress correlates with stunted development of pollen mother cells and abnormal decomposition of the tapetum, decreasing pollen activity [11, 12]. Treatment at 39 °C reduced spikelet fertility in *japonica* by disrupting tapetum functions required for pollen adhesion [13]. High temperatures inhibit the transport of sugars to pollen, reducing activity levels [14]. With reduced spikelet fertility and development under heat stress, rice yield declined seriously [13, 15]. In recent years, significant efforts have been made to dissect the genetic mechanisms of heat tolerance in rice. Many QTLs/genes for heat tolerance at different reproductive stages have been reported using mutants or different genetic populations. Chen et al. mapped *qHTT8* using RIL from heat-tolerant variety HHZ and heat-sensitive variety 9311 [16]. A total of 12 QTLs associated with heat tolerance at booting stage were detected in chromosomal segment substitution lines derived from Sasanishiki and Habataki, and *qHTB4-2* was identified in two environments [17]. Cao et al. mapped six QTLs for heat tolerance in a doubled haploid (DH) population from an *indica*-*japonica* cross of rice (*Oryza sativa* L), with QTLs distributed on chromosomes (Chr) 1, 3, 4, 8, and 11 [18]. In addition to QTLs, some heat-tolerant genes have been identified. *TT3.1* was identified as a putative thermos sensor, and *TT3.1* and *TT3.2* interact to enhance rice thermo-tolerance and reduce grain-yield losses caused by heat stress [19]. *HTH5* encodes pyridoxal phosphate homeostasis protein that can increase the seed-setting rate of rice plants under heat stress at the heading stage [5]. *OgTT1* encodes a proteasome α2 subunit gene that protects cells from heat stress by efficient elimination of cytotoxic denatured proteins and better maintenance of heat-response processes [20].

High temperatures also seriously affect rice chalkiness [21], amylose content [7], gel consistency [22], and gelatinization temperature [8]. High temperatures affect starch and protein storage by reducing the amount of large mature amyloplasts in the endosperm and increasing the number of small immature amyloplasts [23]. In a certain temperature range, the amylose content of high amylose rice varieties increased with increasing temperature, while the amylose content of medium and low amylose varieties decreased with increasing temperature [24]. High temperature (35 °C) reduces grain weight, amylose content, and flour gel consistency [11] and also increased the gelatinization temperature [25]. Previous studies have shown that *CHALK5* controls rice chalkiness [26], *WX* regulates amylose content and gel consistency [6], and *ALK* influences gelatinization temperature [27]. It is important to determine the correlation of quality genes and heat-tolerant QTLs/genes under heat stress.

Some studies have explored the relationship between yield and grain exterior quality under high temperature stress. NIL-*TT2*^*HPS32*^ haplotype showed significantly better seed setting rate and 1,000-grain weight than NIL-*TT2*^*HJX*^ haplotype [9]. Expression of rice soluble starch synthase I in wheat reduced grains of greater weight during heat stress [28]. However, there have been few studies testing combinations of yield and quality genes for use in heat-tolerant rice breeding.

In this study, we genotyped a large panel of 517 rice cultivars and investigated RHSR and chalkiness degree (HCD), amylose content (HAC), gel consistency (HGC), and gelatinization temperature (HGT) under heat stress. The results showed that heat stress seriously influenced seed setting rate and seed quality. According to the results, 16 strong heat-tolerant accessions with greater than 70% RHSR under heat stress were selected for improving heat tolerance breeding in rice. Heat-tolerant quantitative trait loci (QTL) were explored in the full population and a few *indica*-specific QTLs were examined in the *indica* population. To investigate the breeding value of the QTLs, we analyzed polymerization of alleles of the QTLs to improve RHSR and quality of rice under heat stress and then verified the findings in a cross-validation population. Our results revealed that accessions containing at least two SA were required to maintain both the rice yield and high quality under heat stress. To confirm the stable loci *qHTT4.2*, found both in *indica* and *japonica*, we also performed candidate gene analysis. Our findings can facilitate further gene discovery and effective breeding of heat-tolerant and superior-quality at the reproductive stage in rice.

## Results

### Heat-tolerant germplasm with high seed setting rate under heat stress, wide geographical distribution, and great genetic diversity in ***indica*** and ***japonica*** rice

**Fig. 1:**
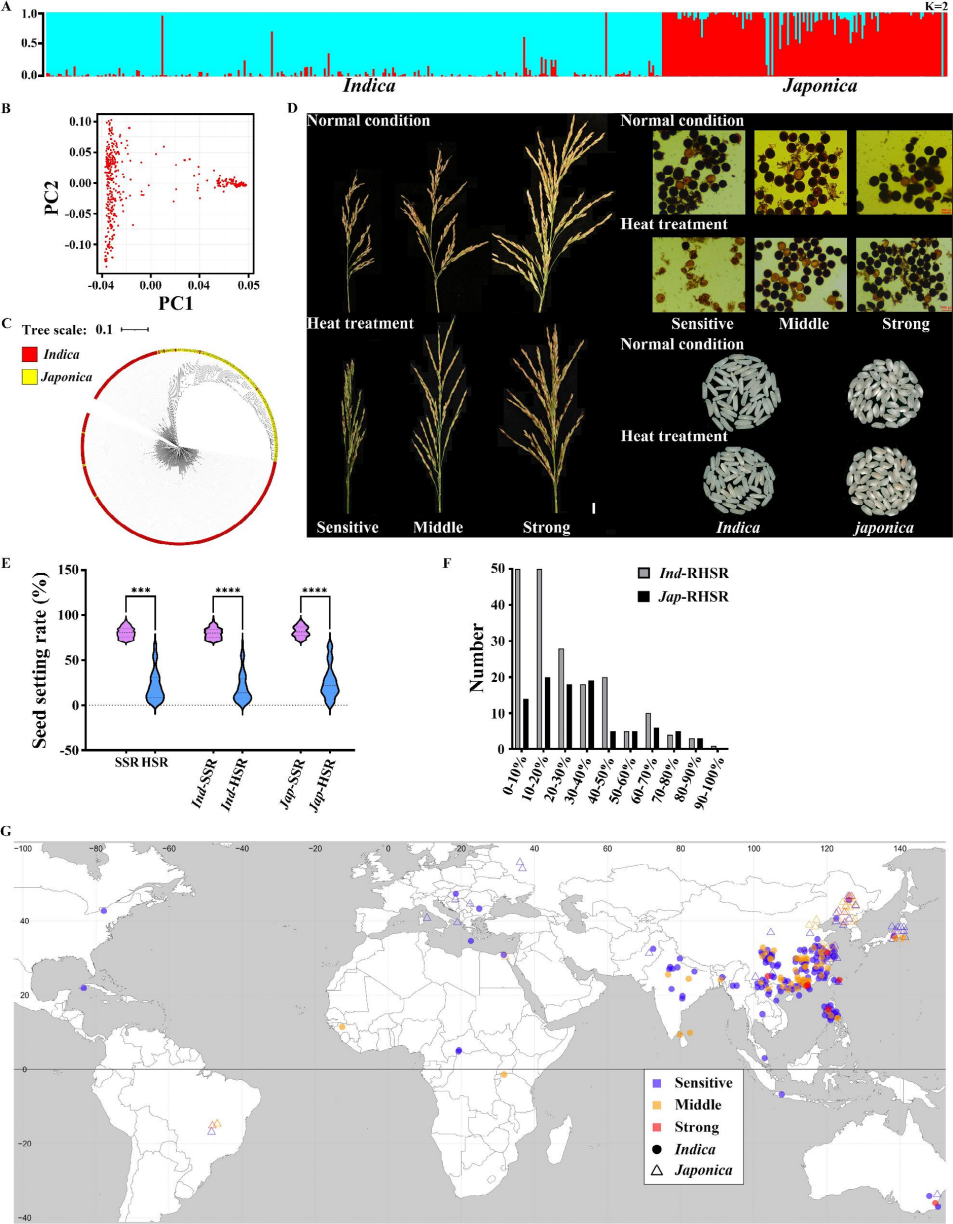
Population structure analysis, phenotypic analysis, Geographical distribution of germplasm. (**A**) The structure of germplasm population. K = 2. (**B**) Principal Component Analysis (PCA). (**C**) NJ tree of germplasm population. (**D**) Change of seed setting rate, pollen fertility of sensitive, middle, strong heat tolerance germplasm and appearance quality of *indica* and *japonica* under heat stress. Bar of spike = 1 cm, Bar of pollen = 500 px. (**E**) Phenotypic analysis of different seed setting rate to full population, *indica* and *japonica*. (**F**) Distribution of RHSR in *indica* and *japonica*. (**G**) Geographical distribution of germplasm with sensitive, middle and strong heat tolerance in *indica* and *japonica*

To evaluate the effects of heat stress at rice flowering stage, the phenotypic characteristics of 517 germplasms from the 3000 Rice Genome Project (3 K-RGP) were determined with or without heat treatment [29] (Table S1). The population structure revealed that panel included *indica* and *japonica* accessions (Fig. 1A). We constructed neighbor-joining trees and performed principal components analysis (PCA) using evenly distributed single-nucleotide polymorphisms (SNPs) (Fig. 1B, C). The results indicated that the population exhibits wide genetic and geographical diversity that is required to identify heat-tolerant germplasms and heat-tolerant loci. Heat stress reduces rice yield because high temperature effects on pollen fertility, chalkiness, and amylose content reduce seed setting rate and quality (Fig. 1D). To select heat-tolerant germplasms for breeding, 284 germplasms including 189 *indica* and 95 *japonica* with seed setting rates higher than 70% under normal conditions were subjected to a genome-wide association study (GWAS) at rice flowering stage to measure heat tolerance. The average seed-setting rate under heat stress (HSR) (21.64%) were lower than the average seed setting rate (SSR) (79.92%) under normal conditions for the 284 germplasms (Table 1; Fig. 1E). The results showed that heat stress seriously decreased the rice yield by reducing SSR. The average and relative seed setting rates were decreased after heat stress treatment of *indica* and *japonica* populations (Table 1; Fig. 1F). And it was found that among the *indica* rice cultivars, 67.72% of the germplasms were classified as heat-sensitive, while 28.04% exhibited moderate sensitivity. Among the *japonica* rice cultivars, 54.73% were found to be heat-sensitive, with 36.84% exhibiting moderate sensitivity. It is worth noting that eight cultivars from each of the *indica* and *japonica* rice germplasms demonstrated strong heat tolerance (Table S2). The results further showed that most of the *indica* or *japonica* germplasms were sensitive to heat stress during the flowering stage, with only a few germplasms exhibiting strong heat tolerance and subsequent quality improvement.

**Table 1:** Seed setting rate under normal condition and heat stress in different population

Population	SSR (%)	HSR (%)	RHSR (%)
ALL	79.92	21.64	27.06
*Indica*	79.30	19.64	24.88
*Japonica*	81.15	25.61	31.38

Heat stress at the flowering stage is a crucial factor in determining rice yield by reducing spikelet fertility. Therefore, identifying germplasms with strong heat tolerance and high SSR and RHSR under heat stress can be beneficial for regional rice breeding. To identify the source of germplasms with heat tolerance, germplasms with different heat tolerances were mapped geographically. Most germplasms in this study were obtained from Southeast Asia. In China, some germplasms of *indica* and *japonica* were derived from moderate-lower reaches of the Yangtze River region (Fig. 1G). The results showed wide distributions of the heat-tolerant germplasms both in *indica* and *japonica.* Germplasms of *indica* with strong heat tolerance were found in Guangdong, Yunnan, Taiwan, and Jiangsu of China and the Philippines near the equator (Fig. 1G). Germplasms of *japonica* with strong heat tolerance came from Heilongjiang of China, and Japan (Fig. 1G). This suggests that the genetic mechanisms of heat tolerance between *indica* and *japonica* is different. The geographical patterns observed indicated that different climate conditions acted as a driving force to promote subspecies differentiation and heat tolerance.

### QTLs in the full population and ***indica*** population and overlapping QTLs responsible for RHSR

**Fig. 2:**
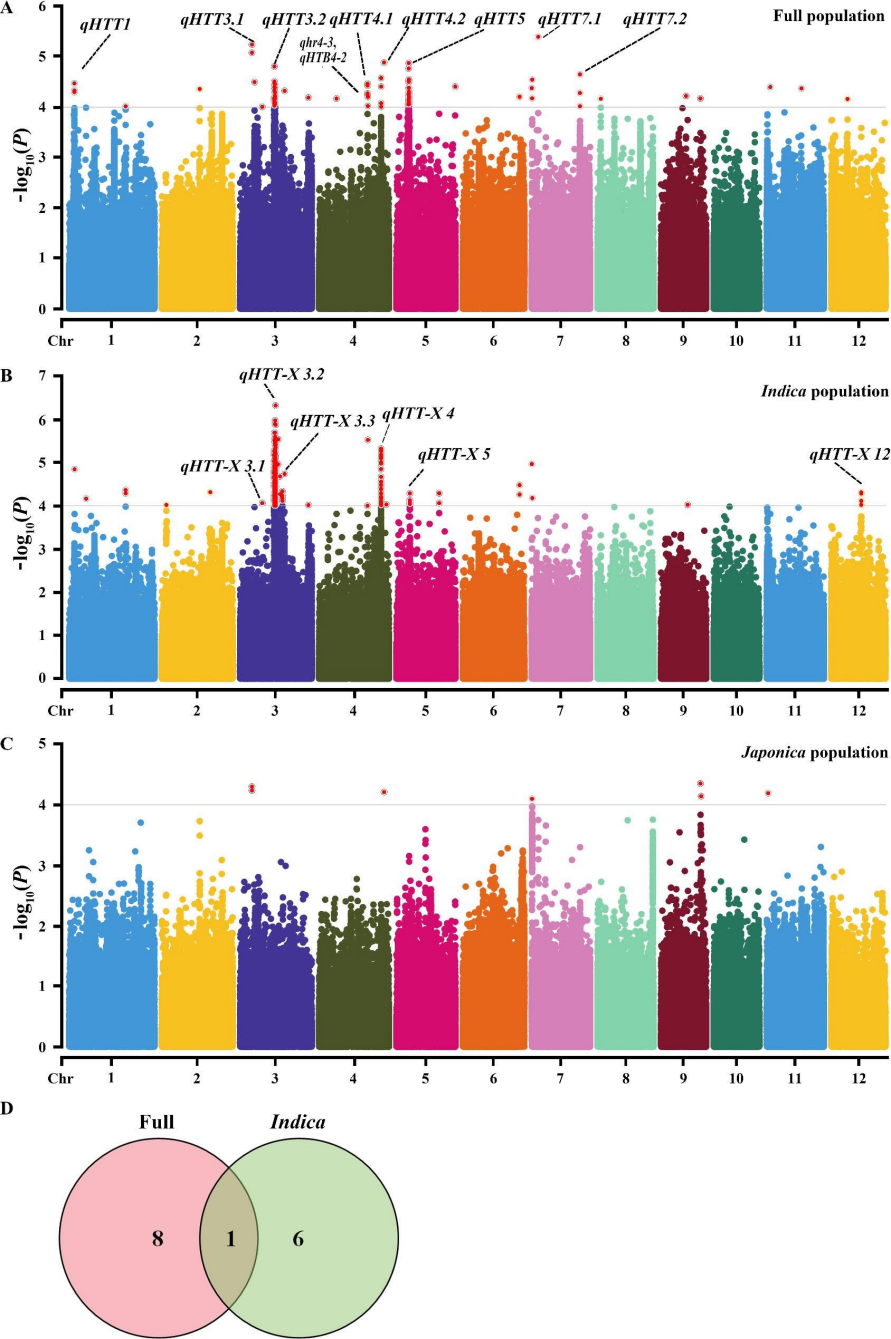
GWAS for heat tolerance at the flowering stage in association panels. **A–C** Manhattan plots of GWAS in the full population **(A)** and *indica***(B)**, *japonica***(C)**. *qhr4-3*, *qHTB4-2* represent associated loci overlapping with reported QTLs and genes, respectively. (**D**) Venn diagram showing unique and shared loci identified in different populations

To explore heat-tolerant genes/QTLs associated with heat tolerance, a GWAS approach was used with RHSR in the full population. The significance threshold of *P* < 0.0001 was set based on the permutation tests, and the population structure and relationships were controlled efficiently (Fig. S1). The analysis of the full population identified eight QTLs, which included 174 significant SNPs related to heat tolerance. These QTLs were distributed on chromosomes 1, 3, 4, 5 and, 7 (Fig. 2A; Table 2). Among them, chromosomes 3, 4, and 7 each contained two QTLs. The QTL *qHTT3.1* on chromosome 3 included 17 SNPs for about 5.7% of the total phenotypic variation explained (PVE) with the most significant signal SNP in the heat stress response (Fig. 2A; Table 2). These significant SNPs included two previously published QTLs, *qHTT3.1* was close to *qhr3-1*, while *qHTT4.2* and *qHTT-X4* were close to *qHTB4-2* [17] (Fig. 2A, B). Taken together, these results suggested that there were many valuable heat-tolerant genes that had yet to be cloned and utilized.

**Table 2:** Association signals for different rice RHSR with strong LD

Name	Signal under FarmCPU	Known gene	MAF	Alleles	Elite allele	log (*P*) under MLM		Mean *R*^*2*^
						Full population	*Indica*	
*qHTT1*	Chr1_2646045	*OsNTL3*	0.31	T/C	C	3.38	-	2.50
*qHTT3.1*	Chr3_17441838		0.32	T/C	C	1.56	4.20	3.40
*qHTT3.2*	Chr3_17758466		0.34	T/A	A	3.51	7.45	3.00
*qHTT4.1*	Chr4_24524161		0.17	G/A	A	3.45	-	2.60
*qHTT4.2*	Chr4_31476593		0.3	G/C	G	2.65	9.70	2.60
*qHTT5*	Chr5_6246272		0.08	C/T	C	1.37	-	3.20
*qHTT7.1*	Chr7_252305		0.14	G/A	A	2.86	-	2.70
*qHTT7.2*	Chr7_24472878		0.12	C/A	A	2.24	-	3.20
*qHTT-XI3.1*	Chr3_18061266		0.04	T/C	C	4.75	-	6.40
*qHTT-XI3.2*	Chr3_20328744		0.05	C/T	T	-	2.08	4.60
*qHTT-XI3.3*	Chr3_21497502		0.10	T/C	C	-	4.59	4.05
*qHTT-XI4.1*	Chr4_31453877		0.30	A/G	A	-	5.37	33.60
*qHTT-XI 5*	Chr5_6820123		0.05	G/A	A	-	5.10	4.10
*qHTT-XI12*	Chr12_15207279		0.17	G/A	A	-	4.88	3.40

To investigate subpopulation-specific association signals for RHSR in *indica* and *japonica*, respectively, we performed GWAS within two subpopulations to explore subpopulation-specific association signals (Fig. 2B, C; Fig. S1). We detected six QTLs in the *indica* populations (Table 2), with three on Chr3, one on Chr4, one on Chr5, and one on Chr12 (Fig. 2B). QTL *qHTT4.2* was an overlapping QTL found both in the full population and in *indica* (Fig. 2C, D). No QTL was detected in the *japonica* population. The results indicated that the full population QTLs and the *indica*-specific QTLs were responsible for heat tolerance.

### More than two SA were accumulated that can enhance RHSR

**Fig. 3:**
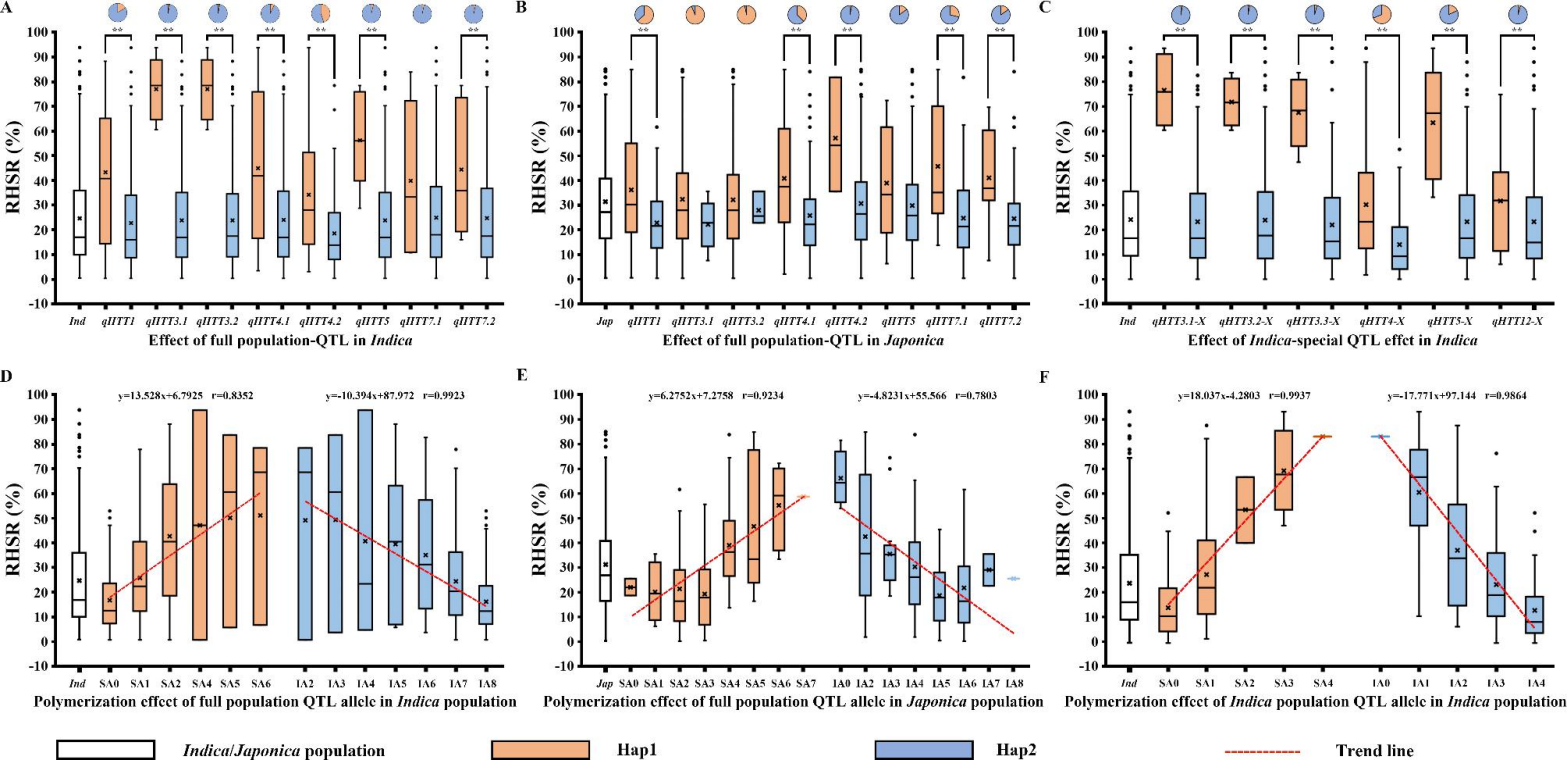
Effect (A–F) and pyramiding effect (G–L) of SA and IA of heat tolerance QTL for RHSR. (**A–B**) Effect of SA and IA of full population heat-tolerant QTL for RHSR in *indica***(A)**, *japonica***(B)**. **C** Effect of SA and IA of *indica-*specific heat-tolerant QTL for RHSR in *indica.***D–E** Pyramiding effect of SA and IA of full population heat-tolerant QTL for RHSR in *indica***(D)** and *japonica***(E)**. (**F**) Pyramiding effect of SA and IA of *indica-*special population heat-tolerant QTL for RHSR in *indica*

To identify allele effects and pyramiding effects of QTLs with RHSR, haplotype analysis was performed using the GWAS and validation populations. Two haplotypes were identified in the GWAS panel. We found that Hap1 was a minority, and the ratio of haplotype2 (Hap2) is much higher than Hap1. (Fig. 3A). The RHSR of Hap1 of *qHTT1*, *qHTT3.1*, *qHTT3.2*, *qHTT4.1*, *qHTT4.2*, and *qHTT7.2* was significantly higher than the RHSR of Hap2 in *indica* (Fig. 3A). Hap1 of q*HTT1*, *qHTT3.1*, and *qHTT3.2* was mainly found in *japonica* (Fig. 3B) and Hap2 of *indica*-specific QTL mainly occurs in *indica* (Fig. 3C). The RHSR of *qHTT3.1*-X, *qHTT3.2*-X, *qHTT3.3*-X, *qHTT4*-X, *qHTT5*-X, and *qHTT12*-X was significantly higher in *indica* rice with Hap1 than that in *indica* rice with Hap2 (Fig. 3C). These findings indicate that the differentiated RHSRs between rice with Hap1 and Hap2 may be caused by different haplotypes of QTLs. To further verify whether accessions with Hap1 have better heat tolerance than those with other haplotypes, we compared the RHSRs among accessions with the same haplotypes of heat-tolerant QTLs but different validation populations. The results showed that, in both *indica* and *japonica*, accessions with Hap1 of *qHTT1*, *qHTT3.1*, *qHTT4.1*, *qHTT4.2*, *qHTT7.1*, and *qHTT7.2* had higher RHSRs than those with Hap2. Furthermore, accessions with Hap1 of *qHTT3.1-X*, *qHTT3.2-X*, *qHTT4-X*, and *qHTT5-X* showed higher RHSRs in the *indica* validation population (Fig. S2C). These results suggested that Hap1 may be a heat-tolerant superior allele. Therefore, we speculate that Hap1 of heat-tolerant QTLs, which enhanced RHSR under heat stress, might be useful resource for improving heat tolerance in rice.

To estimate the potential for breeding utilization of SA of QTLs, we performed combined haplotype analysis for heat tolerance. Results showed that, in *indica*, pyramiding of more than two SA leads to enhance RHSR (*r* = 0.8352), while combining more than four inferior alleles (IA) results in reduced RHSR (*r* = 0.9923) (Fig. 3D). In *japonica*, enhanced RHSR was observed with pyramiding of more than four SA (*r* = 0.9234), and reduced RHSR was found with more than two IA (*r* = 0.7803) (Fig. 3E). We also found that the RHSRs of accessions increased as SA increased (*r* = 0.9937) and decreased as IA increased (*r* = 0.9864) in the *indica* population (Fig. 3F). All germplasms with more than two SA contained *qHTT4.1* and 75% of germplasms also included *qHTT1*. To further confirm the pyramiding effects of heat-tolerant QTLs, we performed haplotype analysis in a cross-validation population. RHSR of accessions with two SA were higher than accessions with one or no SA in *indica* cross-validation population (Figure S2D–F). Similarly, RHSRs of accessions with two more full population SA were higher than those of accessions with one or no SA in *japonica* cross-validation population (*r* = 0.8543) (Figure S2E). Under heat stress, varieties with more than two SA generally exhibited significant RHSRs (Fig. 3F), indicating that the polymerization of SA can be used to enhance resistance of heat stress. *indica* accession contained at least two heat-tolerant SA with average RHSR greater than 43%. Moreover, more than 48% RHSR by pyramiding *qHTT1* and *qHTT4.1* in *indica*, while 43% RHSR by pyramiding *qHTT1*, *qHTT3.1*, *qHTT3.2*, and *qHTT7.1* in *japoica*. (Table S3). Comprehensively, the RHSR of germplasm increased with pyramiding SA or decreased with pyramiding IA. These results suggest that RHSRs are controlled by the additive effects of causal genes. Some common association QTLs (*qHTT4.1*, *qHTT-X4*) showed significant RHSR and pyramiding effects, which could be useful for introducing SA to improve heat tolerance in rice breeding.

### QTLs of RHSR are related to improved yield by interaction with quality genes to balance yield and quality under heat stress at flowering stage

We tested allele effects and pyramiding effects of haplotype combination of heat-tolerant QTLs and quality genes of *CHALK5*, *WX*, and *ALK*. *CHALK5* was found to regulate chalkiness degree [26], *WX* was found to influence amylose content and gel consistency [6], and *ALK* was found to affect gelatinization temperature mainly [27]. RHSR, chalkiness degree (HCD), amylose content (HAC), gel consistency (HGC), and gelatinization temperature (HGT) under heat stress were measured to determine the effect of polymerization of haplotype of heat-tolerant QTLs and haplotype of *CHALK5*, *WX*, and *ALK* combinations in the *japonica* and *indica* populations.

To estimate the potential relationship between heat-tolerant QTLs and *CHALK5*. We analyzed chalkiness degree under normal condition and heat stress with heat-tolerant QTL and *CHALK5* or *chalk5*. Most of the heat-tolerant QTL/*CHALK5* showed no significant differences between normal condition and heat stress, except for *qHTT4.2*/*CHALK5* and *qHTT3.3-X*/*CHALK5* (Table S3). With *chalk5*, heat-tolerant QTL/*chalk5* showed significant differences between normal condition and heat stress (Table S3).

**Fig. 4:**
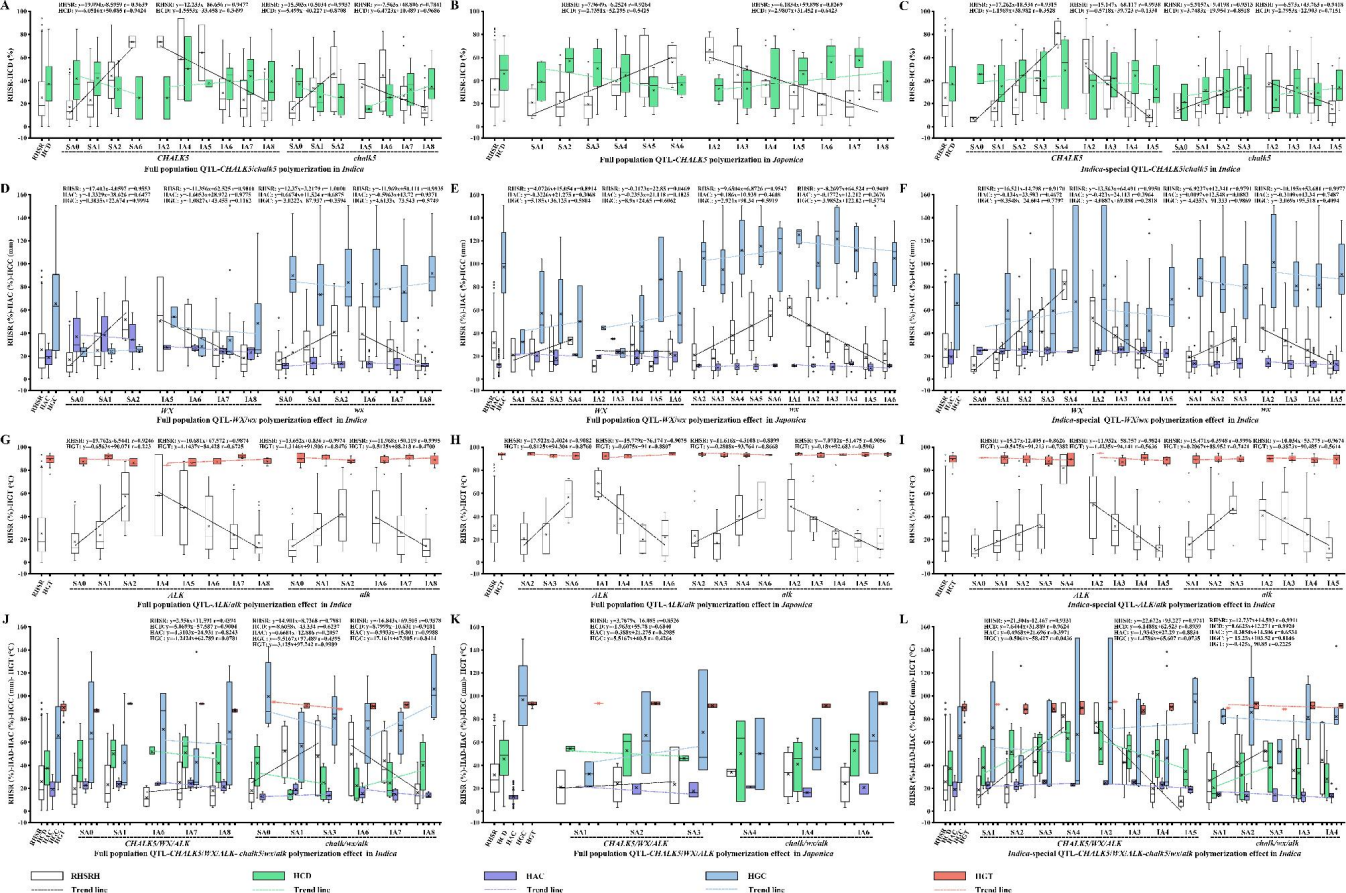
Comparison of superior allele and IA pyramiding and phenotypic performance of RHSR, chalkiness degree (HCD), amylose content (HAC), gel consistency (HGC), gelatinization temperature (HGT) under heat stress. (**A**) RHSR and HCD of SA and IA of full population heat-tolerant QTL with different *CHALK5* haplotype in *indica*. (**B**) RHSR and HCD of SA and IA of full population heat-tolerant QTL with different *CHALK5* haplotype in *japonica*. (**C**) RHSR and HCD of SA and IA of *indica-*specific heat-tolerant QTL with different *CHALK5* haplotype in *indica*. (**D**) RHSR, HAC and HGC of SA and IA of full population heat-tolerant QTL with different *WX* haplotype in *indica*. (**E**) RHSR, HAC and HGC of SA and IA of full population heat-tolerant QTL with different *WX* haplotype in *japonica*. (**F**) RHSR, HAC and HGC of SA and IA of *indica-*special heat-tolerant QTL with different *WX* haplotype in *indica.* (**G**) RHSR and HGT of SA and IA of full population heat-tolerant QTL with different *CHALK5* haplotype in *indica*. (**H**) RHSR and HGT of SA and IA of full population heat-tolerant QTL with different *CHALK5* haplotype in *japonica*. (**I**) RHSR and HGT of SA and IA of *indica-*specific heat-tolerant QTL with different *CHALK5* haplotype in *indica*. (**J**) RHSR, HCD, HAC, HGT of SA and IA of full population heat-tolerant QTL with *CHALK5*/*WX*/*ALK* and *chalk5*/*wx*/*alk* in *indica*. (**K**) RHSR, HCD, HAC, HGT of SA and IA of full population heat-tolerant QTL with *CHALK5*/*WX*/*ALK* in *japonica*. (**L**) RHSR, HCD, HAC, HGT of SA and IA of *indica* population heat-tolerant QTL with *CHALK5*/*WX*/*ALK* and *chalk5*/*wx*/*alk* in *indica* Note: Dotted line means trends of phenotypic change. Formula means the fitting degree of regression line to the observed value

With the accumulation of SA of heat-tolerant QTLs, the RHSRs of the varieties increased with *CHALK5* or *chalk5* in both the *indica and japonica* populations (Fig. 4A, B, C). We then investigated the HCD of *CHALK5* and *chalk5* with SA and found that *chalk5* controlled lower HCD than *CHALK5* with any SA in *indica* (Fig. 4A). As the number of SA (*r* = 0.9639) increased, the HCD of varieties declined in *indica* with *CHALK5* (r = 0.9424)/*chalk5* (r = 0.8708) (Fig. 4A). Correspondingly, in the *japonica* population, with the accumulation of SA, the RHSRs of the varieties increased with *CHALK5* (*r* = 0.9264) (Fig. 4B). HCD (*r* = 0.5425) decreased with accumulation of SA with *CHALK5* (Fig. 4B). In the *indica* population, RHSRs of the accessions increased with polymerization of SA (*r* = 0.9315, *r* = 0.9313) and decreased with polymerization of IA (*r* = 0.9938, *r* = 0.9418) with *CHALK5* and *chalk5* (Fig. 4C). With the polymerization of SA of QTLs, the RHSRs of the varieties increased with *CHALK5* or *chalk5* in *indica* cross-validation (Fig. S2A–C). HCD increased with polymerization of SA with *CHALK5* in *indica* cross-validation (Fig. S2A). The results showed a positive regulation of HCD by SA polymerization. The accessions with more than two full population QTLs SA showed significantly higher RHSRs under *CHALK5* or *chalk5*.

With the accumulation of more than two SA, the RHSRs of the varieties increased with *WX* or *wx* in *indica* and *japonica* (Fig. 4D–F). Correspondingly, in the *japonica* population, with the accumulation of SA, the RHSRs of the varieties increased with *WX* (*r* = 0.9553) or *wx* (*r* = 1.0) and HGC decreased in *japonica* with SA polymerization with *WX* or *wx* (Fig. 4D). As the number of SA increased, the HAC of varieties increased in *indica* with *WX* or *wx* (Fig. 4D–F). As the number of SA increased, the HGC of varieties increased in *indica* with *WX* (*r* = 0.9994, *r* = 0.7797) but the number of SA increased, the HGC of varieties declined in *indica* with *wx* (*r* = 0.5919, *r* = 9869) (Fig. 4D, F). As the number of IA increased, the HGC of varieties lengthened in *indica* with *wx* (*r* = 0.5748, *r* = 0.4094) (Fig. 4D–F). With the polymerization of SA of QTLs, the RHSRs of the varieties increased with *WX* or *wx* in *indica* cross-validation (Fig. S3D–F). HAC increased with polymerization of SA with *WX* or *wx* in *indica* cross-validation (Fig. S3D–F). HGC of accessions were shortened with polymerization of SA with *WX* or *wx* in *indica and japonica* cross-validation (Fig. S3D–F). The results showed a positive regulation of HAC and negative regulation of HGC by SA polymerization of heat-tolerant QTLs. As accessions with more than two SA achieved 43% RHSR in *indica*, heat-tolerant QTLs offered yield basic for HAC and HGC.

We then investigated RHSRs of heat-tolerant QTLs and HGT of *indica* and *japonica*. Accessions with more SA of QTLs with *ALK*/*alk* showed higher RHSRs in *indica* and *japonica* (Fig. 4G–I). The HGT was decreased with an increased number of SA and decreased number of IA with *ALK* in *indica* and *japonica*, respectively (Fig. 4G–I). *Alk* corresponded to lower HGT than *ALK* under alleles of heat-tolerant QTLs.

With the accumulation of SA, RHSRs of the varieties increased with C*HALK5/WX/ALK* or *chalk5/wx/alk* in *indica* and *japonica* (Fig. 4J–L). HAC of varieties increased gradually with accumulation of SA combined with C*HALK5*/*WX*/*ALK* and *chalk5*/*wx*/*alk* in *indica* (Fig. 4J–L). HAC of varieties was increased gradually with accumulation of IA of QTLs with C*HALK5/WX/ALK* and *chalk5/wx/alk* in *indica* (Fig. 4J–L). HGC of varieties decreased with SA polymerized with *CHALK5*/*WX*/*ALK* and *chalk5*/*wx*/*alk* in *indica* and *japonica* (Fig. 4J–L). These results are similar to those for RHSR, HAC, and HCD in the cross-validation population (Fig. S3G–I). Interestingly, a few accessions contained more than two SA as well as *chalk5*/*wx*/*alk* (Fig. 4A-L). This indicates that accessions can improve yield and quality by coordinating heat-tolerant QTLs and quality genes.

Thirty-eight genotype-Hap1 showed different phenotype. *qHTT1*/*qHTT3.1*/*qHTT3.2*/*qHTT4.1*/*qHTT5*/*qHTT7.2*/*CHALK5* had RHSR of 73.26% and HCD of 24.65% in *indica* which was the highest RHSR (Table S4). In *indica*, *qHTT1*/*qHTT4.1*/*chalk5* had the lowest HCD of 21.99%. In *japonica, qHTT1*/*qHTT3.1*/*qHTT3.2*/*qHTT4.1*/*wx* had an HGC of 136.25 mm, which was the longest HGC (Table S4). *qHTT1*/*qHTT3.1*/*qHTT3.2*/*qHTT5*/*wx* had an HAC of 7.73%, which was the lowest HAC (Table S4). This indicates that multiple genotype-Hap1 can regulate RHSR, HCD, HAC, HGC, and HGT under heat stress. With *CHALK5*/*WX*/*ALK* or *chalk5/wx*/*alk*, the same QTL with hap1 and hap2 had no significant differences in RHSR, HCD, HAC, HGC, and HGT (Table S5).

**Fig. 5:**
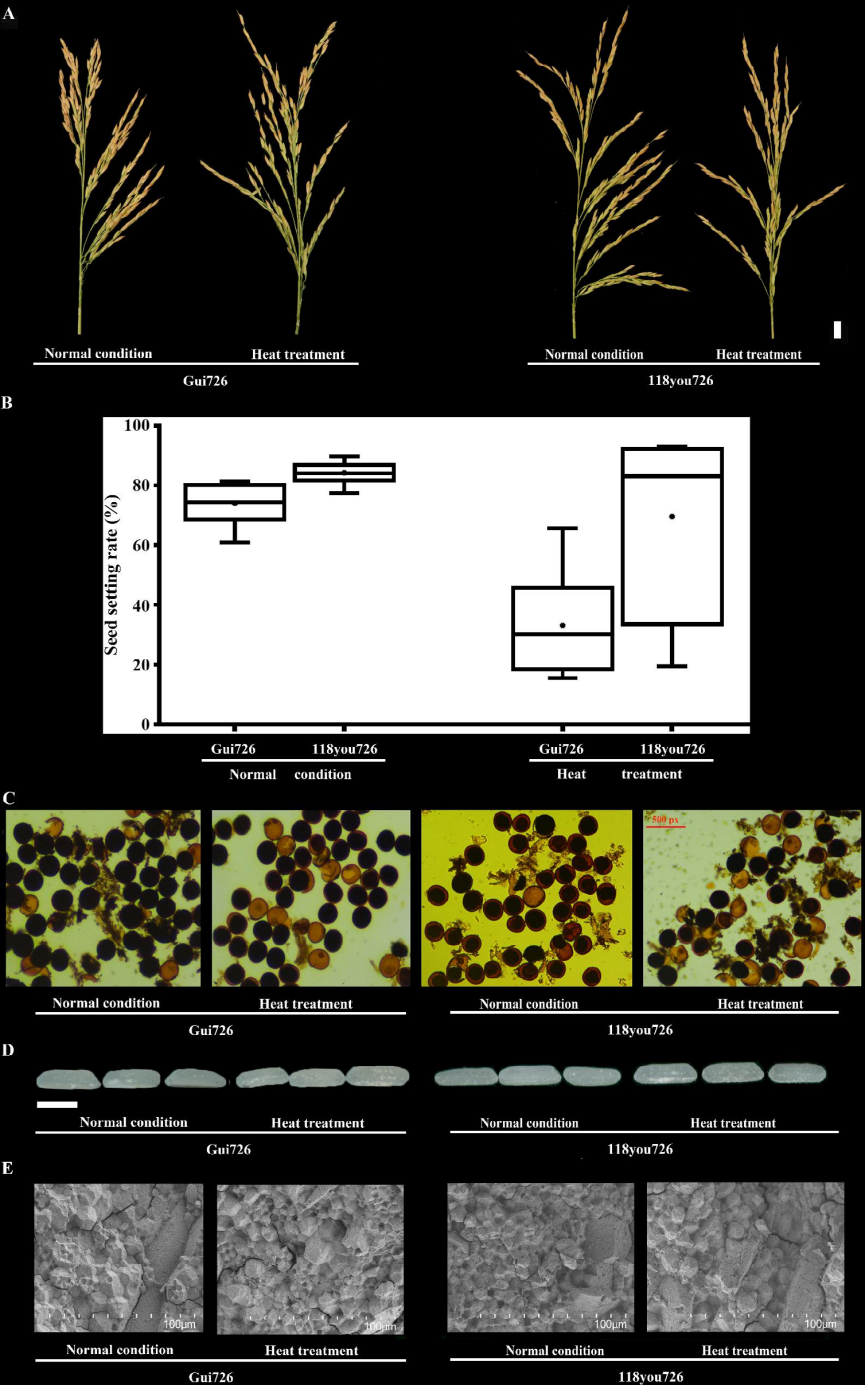
Phenotype of Gui726 and 118you726 under normal condition and heat stress. (**A**) Spike of Gui726 and 118you726 under normal condition and heat stress. Bar = 1 cm. (**B**) Seed setting rate of Gui726 and 118you726 under normal condition and heat stress. (**C**) Pollen of Gui726 and 118you726 under normal condition and heat stress. Bar = 500 px. (**D**) Exterior quality of Gui726 and 118you726 under normal condition and heat stress. (**E**) Scanning electron microscope images of chalkiness in Gui726 and 118you726 under normal condition and heat stress. Bar = 100 μm

To further clarify whether accessions with heat-tolerant QTLs and quality genes have better heat tolerance and quality, we investigated variety Gui726, with *qHTT4.2*, *qHTT-X-3.1* and *chalk5*, *wx*, and *alk* (Fig. 5A). The RHSR of Gui726 was 59.81% (Fig. 5A–C), with low HCD, HAC, and HGT and long HGC (Fig. 5D, E) (Table S6). 118you726, a variety derived from Gui726 and sterile line 118 A, which held *qHTT4.2*, *qHTT-X-3.1*, *chalk5*, *wx*, and *alk*, showed a high RHSR of 69.47% (Fig. 5A–C) and superior quality (Fig. 5D, E; Table S4). Overall, the results suggested that with the accumulation of SA of heat-tolerant QTLs, the RHSRs of the varieties increased with different haplotypes of *CHALK5*, *WX*, and *ALK*. QTLs of RHSR interact with quality genes. To meet the goals of breeding and obtaining specific genotypes of varieties, yield and quality can be balanced by choosing SA of heat-tolerant QTLs and *chalk5*, *wx*, and *alk* combinations under heat stress.

### ***qHTT4.2*** enhancing RHSR and balancing quality genes

**Fig. 6:**
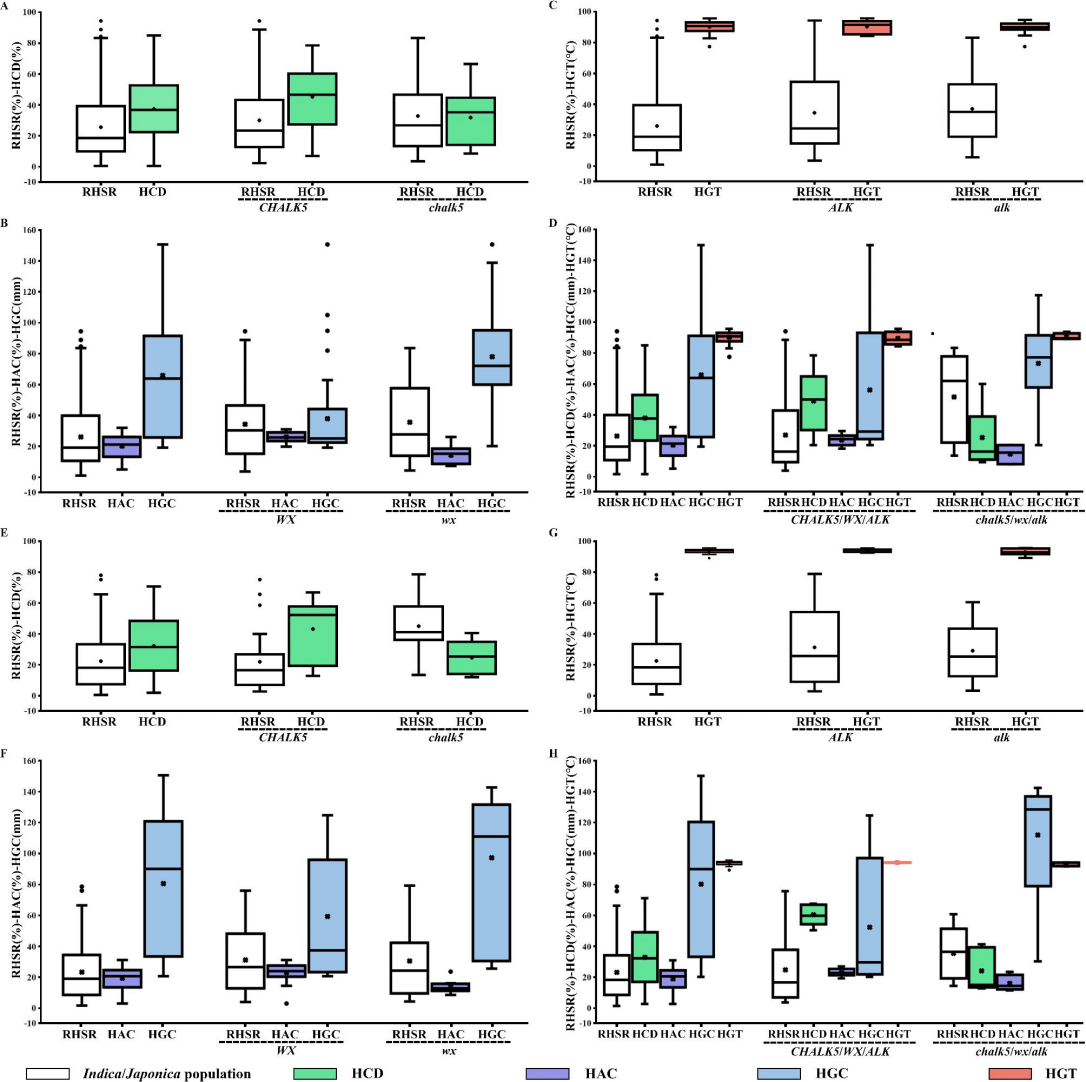
RHSR, HCD, HAC, HGT of SA of *qHTT4.2* with different *CHALK5*, *WX*, *ALK* haplotype and candidate gene analysis. (**A**) RHSR, HCD of *qHTT4.2-*Hap1 with different haplotype of *CHALK5*. (**B**) RHSR, HAC and HGC of *qHTT4.2-*Hap1 with different haplotype of *WX*. (**C**) RHSR and HGT of *qHTT4.2-*Hap1 with different haplotype of *ALK*. (**D**) RHSR, HCD, HAC, HGT of *qHTT4.2-*Hap1 with *CHALK5*/*WX*/*ALK* and *chalk5*/*wx*/*alk* in *indica*. (**E**) RHSR, HCD of *qHTT4.2-*Hap1 with different haplotype of *CHALK5* in *indica* cross-validation population. (**F**) RHSR, HAC and HGC of *qHTT4.2-*Hap1 with different haplotype of *WX* in *indica* cross-validation population. (**G**) RHSR and HGT of *qHTT4.2-*Hap1 with different haplotype of *ALK* in *indica* cross-validation population. (**H**) RHSR, HCD, HAC, HGT of *qHTT4.2-*Hap1 with *CHALK5*/*WX*/*ALK* and *chalk5*/*wx*/*alk* in *indica* cross-validation population

Among the identified loci, a novel locus *qHTT4.2* at 31.47–31.49 Mb on Chr4 was repeatedly detected with strong signals (Table 1). When combined with *chalk5*, *qHTT4.2-Hap1/chalk5* showed RHSR and HCD of 32.28% and 31.26, respectively (Fig. 6A). The HAC and HGC of *qHTT4.2-Hap1/wx* showed significant differences compared to *qHTT4.2-Hap1*/*WX* (Fig. 6B). Additionally, the HGT of *qHTT4.2-Hap1*/*alk* was lower than that of *qHTT4.2-Hap1/ALK* (Fig. 6C). Notably, RHSRs of *qHTT4.2-*Hap1/*chalk5, qHTT4.2-*Hap1*/wx*, and *qHTT4.2-*Hap1*/alk* were higher than those of *qHTT4.2-*Hap1 combined with *CHALK5*, *WX*, and *ALK* (Fig. 6D). Furthermore, the yield and quality of *qHTT4.2-*Hap1/*chalk5*/*wx*/*alk* showed better performance than *qHTT4.2-*Hap1/*CHALK5*/*WX*/*ALK* (Fig. 6D). These results are similar to those for RHSR, HGT, HAC, and HCD in the cross-validation population (Fig. 6E–H). Overall, the results indicated that *qHTT4.2* had a positive effect in balancing yield and quality under heat stress, and can be used for breeding heat-tolerant rice varieties.

**Fig. 7:**
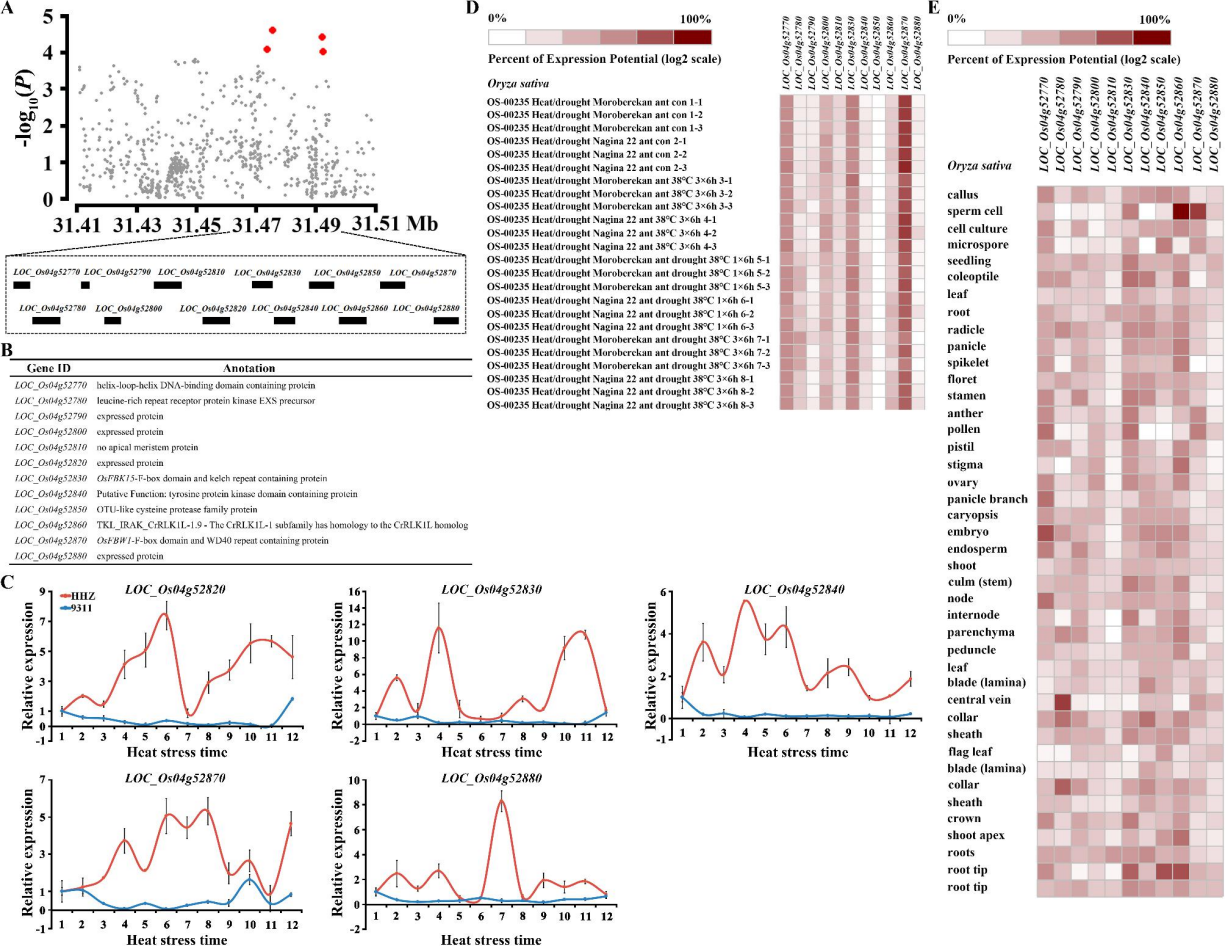
Candidate gene analysis of *qHTT4.2*. (**A**) Local Manhattan plots surrounding the peak of *qHTT4.2* in *indica* population and corresponding genes overviews. Red dots indicate predicted genes possessing significant SNPs. (**B**) Details for 12 potential candidate genes. (**C**) Heat-induced expression analysis of five potential candidate genes in the spikelet at the flowering stage. Data represent means s.d. (n = 3). (**D**) Heat-induced expression analysis of eleven potential candidate genes at the flowering stage in N22. (**E**) Expression analysis of eleven potential candidate genes in tissue, and the gene expression data were acquired from Genevestigator (https://genevestigator.com/)

To determine the underlying candidate genes responsible for the QTLs effects, we performed local linkage disequilibrium (LD) analysis (Fig. 7A) and found that *qHTT4.2* corresponded to a 19 kb interval containing 11 predicted genes (Fig. 7B). We conducted heat-induced expression analysis in the heat-tolerant variety Huanghuazhan (HHZ) and the heat-sensitive variety 9311, which were previously used to study heat stress at the flowering stage [16]. *Loc_Os04g52830* and *Loc_Os04g52870* were highly expressed after heat stress in HHZ. Heat-induced expression analysis showed that *Loc_Os04g52830* and *Loc_Os04g52870* were induced in the spikelets at the flowering stage (Fig. 7C). Tissue expression analysis further demonstrated that *Loc_Os04g52830* and *Loc_Os04g52870* were highly expressed in panicles, anthers, and pollen according to Genevestigator (Fig. 7D). In cross-validation, *Loc_Os04g52830* and *Loc_Os04g52870* showed high expression in the anthers of N22 (a strong heat-tolerant variety) under heat treatment according to Genevestigator (Fig. 7E). In addition, *Loc_Os04g52830*, encoding an *OsFBK15* - F-box domain and Kelch repeat-containing protein, was previously reported to possibly control cold tolerance at the booting stage [30]. Taken altogether, we concluded that *Loc_Os04g52830* and *Loc_Os04g52870* are the most likely candidate genes responsible for *qHTT4.2*.

## Discussion

### Identification of QTLs for heat tolerance by GWAS

Exploring the genetic mechanism of heat tolerance in rice is crucial for the cultivation of heat-resistant rice to adapt to the global warming environment [31]. Chen et al. found one QTL on chromosome 8 with spikelet fertility rate under high temperature stress [16]. And twelve QTLs associated with heat tolerance were detected in a set of Sasanishiki and Habataki chromosome segment substitution lines [17]. Our study found that QTL *qHTT3.1* was close to *qhr3-1*; while *qHTT4.2* and *qHTT-X4* were close to *qHTB4-2*, which corresponds to previous research and provides validation for these findings [17]. In addition, *qSSR6-1*, *qSSR7-1*, *qSSR8-1*, *qSSR9-1* and *qSSR11-1* located on chromosomes 6, 7, 8, 9 and 11 from heat-tolerant *indica* variety N22 and a heat-sensitive *indica* variety 9311 [32]. PS et al. used recombinant inbred lines of N22 and IR64 to map heat-tolerant QTL *qSTIY5.1*/*qSSIY5.2* at the booting stage, which overlapped with previously identified QTLs [33]. Jagadish et al. identified a major QTL explaining 18% of the phenotypic variation using recombinant inbred lines of *indica* Bala and Azucena, which is located in the same region as previously mapped QTLs [34]. Overall, these findings demonstrate the value of heat tolerance QTLs in breeding heat-tolerant rice varieties.

### Yield and quality can be balanced under high temperature stress by polymerizing the main heat-tolerant and quality genes

High temperature stress threatens rice production by reducing spikelet fertility during the flowering stage [5]. High temperature can also negatively impact rice chalkiness [21], amylose content [7], gel consistency [22], and gelatinization temperature [8]. In response to heat stress, rice plants trigger a cascade of complex transcriptional regulatory networks [35]. High temperature causes physiological effects including membrane damage [36], reactive oxygen species accumulation [37], photosynthesis damage [38], disturbance of carbohydrate metabolism and partitioning [15], and phytohormone imbalance [39]. Rice can sense heat stress and induce signal cascades [40] and transcriptional regulatory networks regulating heat stress responses and protein homeostasis [41]. Genes in these pathways can maintain rice production under high temperature stress. *TT3.1* and *TT3.2* interact to enhance rice thermotolerance and reduce grain-yield losses caused by heat stress by protecting chloroplasts [19]. *HTH5* encodes pyridoxal phosphate homeostasis protein and reduces reactive oxygen species accumulation to increase the seed-setting rate [5]. The cooperation of heat-tolerant genes and quality genes is required for breeding of stress resistance in rice [42].

There are abundant genetic resources that could be used for breeding to improve multiple traits. For example, Schulthess et al. collected and analyzed genetic profiles evaluated grain yield and resistance to yellow rust in a large winter wheat collection, and then designed an Elite × PGR’ F_1_ cross [43]. In our study, the heat-tolerant variety Gui726 included a heat-tolerant QTLs and superior quality alleles, with high RHSR and better quality. According to the germplasm genotypes, yield and quality under high temperature stress can be balanced by polymerizing the main heat-tolerant and quality genes during breeding.

### ***qHTT4.2*** can help balance yield and quality under heat stress

The *qHTT4.2* includes two F-box protein genes. The F-box protein family plays various roles in plant hormonal signal transduction and responses to both biotic and abiotic stresses [44]. For instance, the wheat F-Box protein gene *TaFBA1* may improve enzymatic antioxidant levels and can interact with *TaASRP1*, a gene that is involved in plant tolerance to heat stress [45]. *Ctb1* encodes an F-box protein that enhanced cold tolerance in rice at the booting stage [30], while *LTSF1* and *LTSF2* F-box proteins may positively regulate low-temperature stress tolerance by activating antioxidant-enzyme activities in pepper (*Capsicum chinense*) [46]. The F-box protein FBXA-158 promotes thermotolerance and co-immunoprecipitates with CUL-6 in *C. elegans* [47]. *AtPP2-B11* is a key responsive gene to oxidative stress, encoding a Ca^2+^-permeable transporter that links reactive oxygen species [23] and cytosolic Ca^2+^ in *Arabidopsis* [48]. The thermotolerance gene *TT2* senses Ca^2+^ and encodes a transcription factor that mediates Ca^2+^-enhanced interaction with calmodulin [9]. F-box proteins may be involved in hormonal signal transduction under heat stress. Heat stress can cause the accumulation of cellular reactive oxygen species (ROS), and F-Box proteins can also be regulated by heat stress. The F-box gene *OsPP12-A13* can reduce ROS levels and modulate activities of peroxidase and glutathione S-transferase in *Arabidopsis* under abiotic stress [49]. The thermo-tolerant gene *HTH5* reduces the accumulation of ROS at high temperatures by increasing the heat-induced pyridoxal 5’-phosphate content [5]. F-box genes are likely to be involved in signal transduction and ROS processes response to heat stress.

### Conclusions

In this study, heat-tolerant QTLs and genes associated with heat tolerance in rice were identified. The characterization of the effects of heat-tolerant QTLs and quality polymerization can guide future efforts to improve heat tolerance and quality in rice.

### Methods

#### Plant materials

A total of 517 *Oryza sativa* accessions from 3 K-RGP, including landraces of a rice core collection and elite varieties, were used in this study (Table S1). The 517 *Oryza sativa* accessions with average sequencing depth greater than 12 × had clear geographic origin. Information on the ecotype for these rice accessions was obtained from the Catalog of Rice Germplasm Resources in China and IRRI. All the genomic data of 517 rice accessions from the 3 K-RGP can be downloaded from https://aws.amazon.com/public-data-sets/3000-rice-genome/. Study protocol comply with relevant institutional, national, and international guidelines and legislation. The collection included 368 *indica* and 149 *japonica* rice. The sequence data for the rice core collection were obtained from the 3000 Rice Genome Project [50]. A panel of 189 *indica* and 95 *japonica* accessions were used for GWAS and haplotype analysis and 126 *indica* and 38 *japonica* rice were used for cross-validation.

#### Heat stress treatment and phenotyping

All plant materials were cultivated in natural field conditions in the rice paddy field of Rice Research Institute of Guangxi Academy of Agricultural Sciences in Nanning, China, in the summer of 2021. The distance was maintained at 16.7 cm between two plants, whereas distance was maintained at 20 cm between rows. The plants were moved into a phytotron at flowering stage. During this period, the panicles were marked with PVC tags and exposed to 38/28°C day/night temperatures with 6 h (from 09:30 am to 3:30 pm) of 38 °C and the rest at 28 °C during the day in the phytotron (Fig. S4) [16]. After three days of exposure to high temperature, the plants were moved back to the experimental field and the phenotypes were measured. At least three panicles were marked for each plant and at least three plants were subjected to heat stress for each accession. To evaluate yield performance under heat stress, after harvest, the seed setting rate (HSR) was measured for the labeled panicles and panicles were tested for seed setting rate under normal condition (SSR). RHSR was defined as the seed-setting rate under heat stress/seed setting rate under normal conditions. An accession with SSR higher than 70% and RHSR higher than 70% was considered an accession with strong heat tolerance, one with SSR higher than 70% and RHSR between 30 and 70% was considered an accession with moderate heat tolerance accession, and an accession with SSR higher than 70% and RHSR lower than 30% was considered a heat-sensitive accession [51]. Fully filled grains were used to measure the grain chalkiness degree with a rice-quality analyzer (SC-E, Hangzhou Wanshen Test Technology Corporation, China). All trait measurements were repeated at least three times. HAC and HGCs of accession were measured as described previously with slight modification [52, 53]. HGT was measured based on the pasting properties using a rice-rapid visco analyzer method [54]. HAC, HGC, and HGT were measured by Nanjing Origin Testing Technology Corporation in Nanjing, China.

#### GWAS

To select heat-tolerant germplasms for breeding, 284 germplasms including 189 indica and 95 *japonica* with seed setting rates higher than 70% under normal conditions were subjected to a genome-wide association study (GWAS) at the rice flowering stage to evaluate heat tolerance. Totals of 3,851,692 and 3,002,287 high-quality SNPs (MAF ≥ 5%, missing rate < 25%) from Panels 1 and 2, respectively, were used to perform GWAS using the general linear and compressed mixed linear models in the GAPIT package in an R environment [55]. The genome-wide significance threshold was determined by permutation tests with 1000 replications [29]. A region containing more than three consecutive significant SNPs was considered a single associated signal, and the SNP with the minimum *P*-value within the associated signal was considered the lead SNP. For the analysis of candidate genes of the associated signal, the continuous region closely linked to the lead SNP (*R*^*2*^ ≥ 0.6) was considered as the local LD interval [56]. The significance threshold was determined using 0.05/N, where N represents the total number of SNPs. GWAS experiments were performed by Beijing Compass Biotechnology Corporation, Ltd (Beijing, China). Our way to determine the significant threshold is to randomly permute the genotype sample names while keeping both the phenotypes and kinship matrix constant. The top false positive signals under the permutation test with 1000 replications were ranged from 3.5 to 3.8 in *indica*, *japonica* and full population. So, we determined the significant threshold for all three groups was 4.0.

#### Gene genotyping by PARMS

Genotyping of 517 *Oryza sativa* accessions was performed using PARMS (Penta-primer amplification refractory mutation system) according to Pan et al. [57].

#### RNA extraction and qRT-PCR

To analyze gene expression induced by heat stress, HHZ and 9311 plants were subjected to heat stress for 3 days at the flowering stage in the phytotron. RNA samples were collected at 12 different time points [16]. RNA extraction and qRT-PCR were performed as described [58]. Primers were designed using Primer 5.0 (Table S7).

### Statistical analysis

The evaluation of heat treatment was repeated at least three times, and the statistical significance of differences between a population and its parents was determined using one-way ANOVA and Tukey’s post-hoc test. The phenotypic differences were tested using SPSS (IBM Corporation, NY, USA). The gene expression data were acquired from Genevestigator (https://genevestigator.com/).

## Electronic supplementary material

Below is the link to the electronic supplementary material.

Supplementary Material 1

Supplementary Material 2

Supplementary Material 3

Supplementary Material 4

Supplementary Material 5

Supplementary Material 6

Supplementary Material 7

Supplementary Material 8

Supplementary Material 9

Supplementary Material 10

Supplementary Material 11

## Data Availability

The sequencing data of 517 rice accessions used for our analyses can be obtained via project accession PRJEB6180 from NCBI (https://www.ncbi.nlm.nih.gov/sra/?term=PRJEB6180), accession ERP005654 from DDBJ (https://www.ddbj.nig.ac.jp/index-e.html) and from the GigaScience Database (10.5524/200001). All data generated or analyzed during this study are included in this manuscript, its supplementary information files and publicly available repositories.
