# Supplementary figures and images for: Utilization of natural alleles for heat adaptability QTLs at the flowering stage in rice
Source: BMC Plant Biol. 2023 May 16;23:256. doi: 10.1186/s12870-023-04260-5 (PMC10186738; doi:10.1186/s12870-023-04260-5)

A

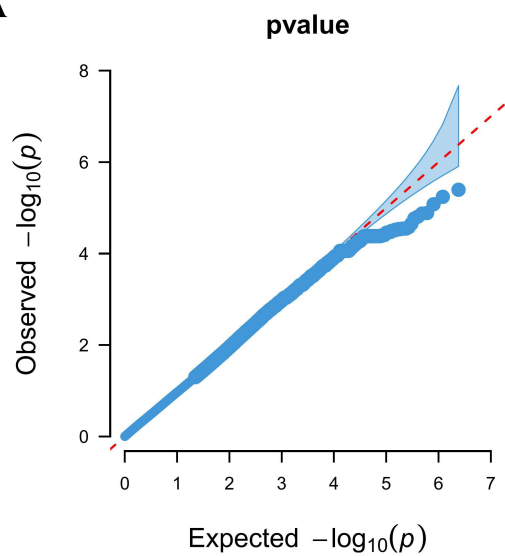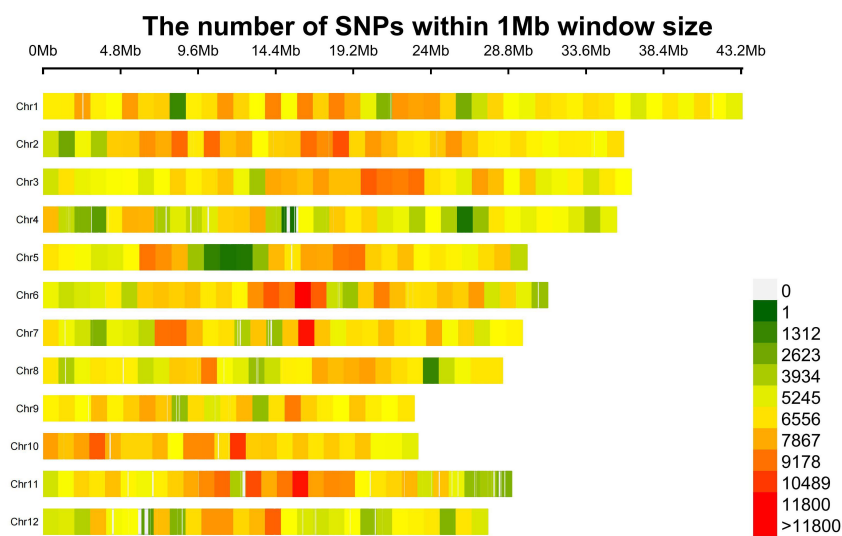

B

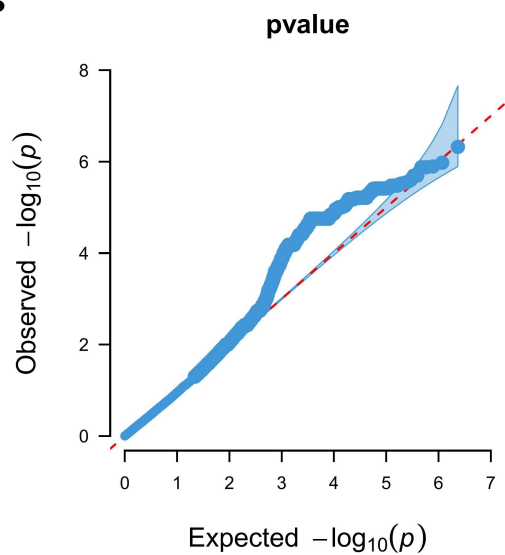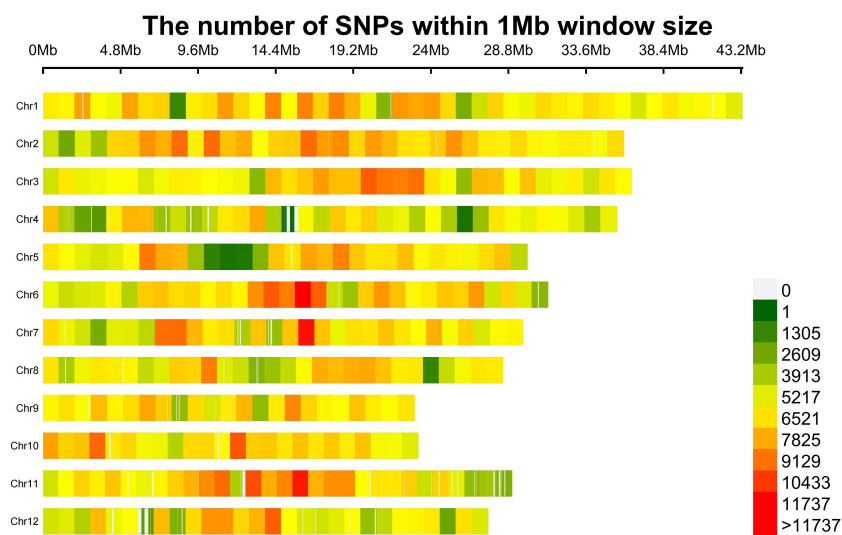

C

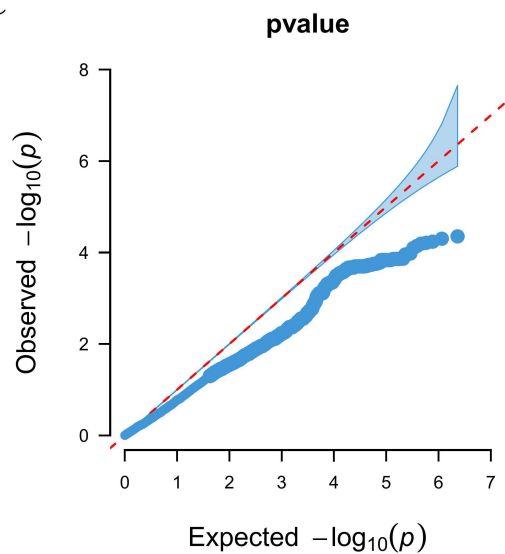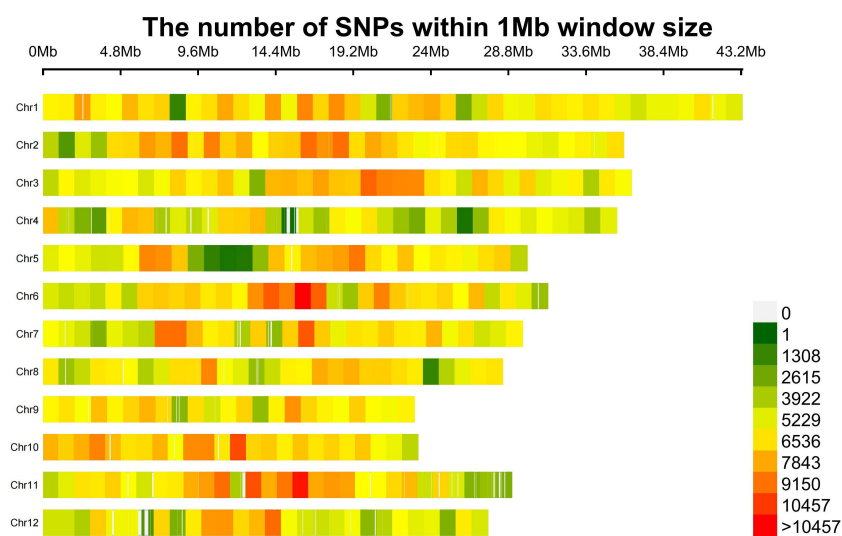

Supplement: Supplementary file 1 — Supplementary Material 1 [file 12870_2023_4260_MOESM1_ESM.pdf]

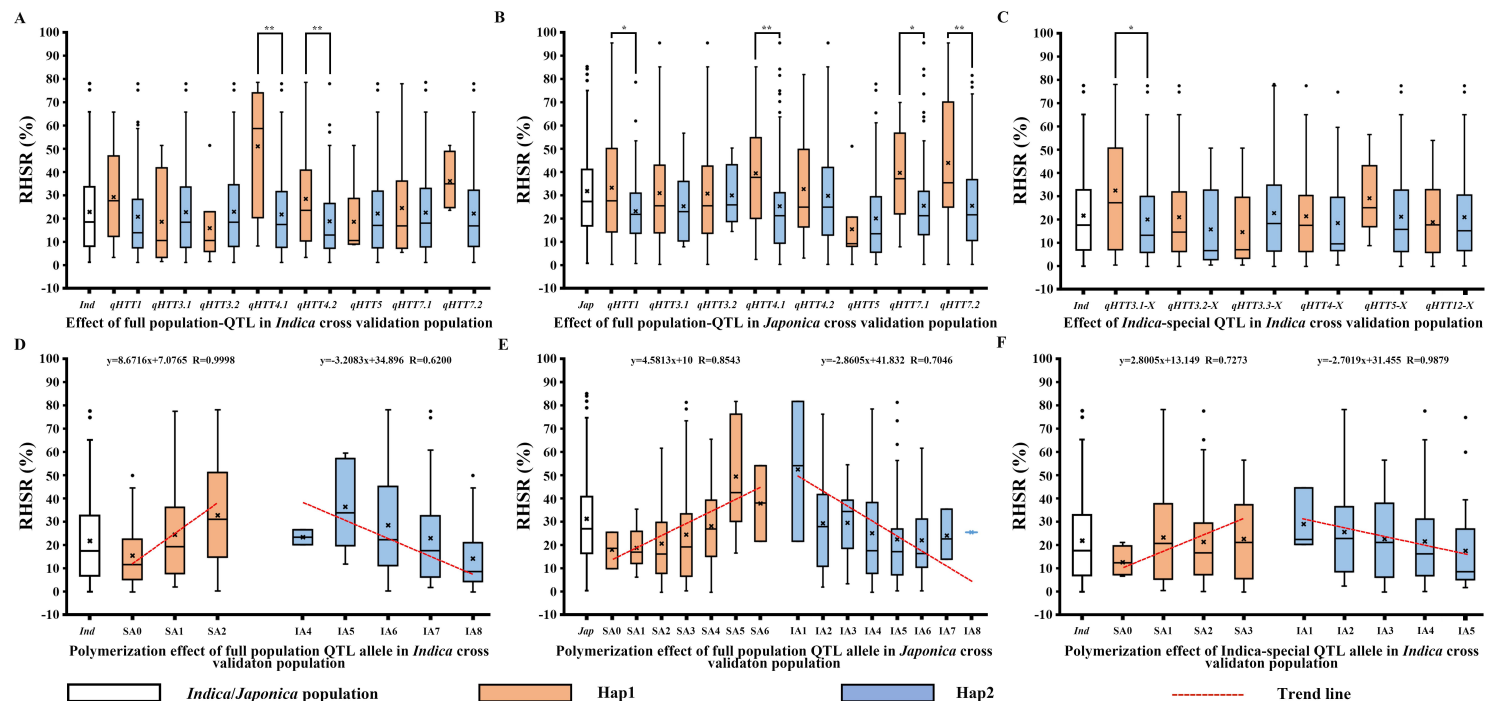

Supplement: Supplementary file 2 — Supplementary Material 2 [file 12870_2023_4260_MOESM2_ESM.pdf]

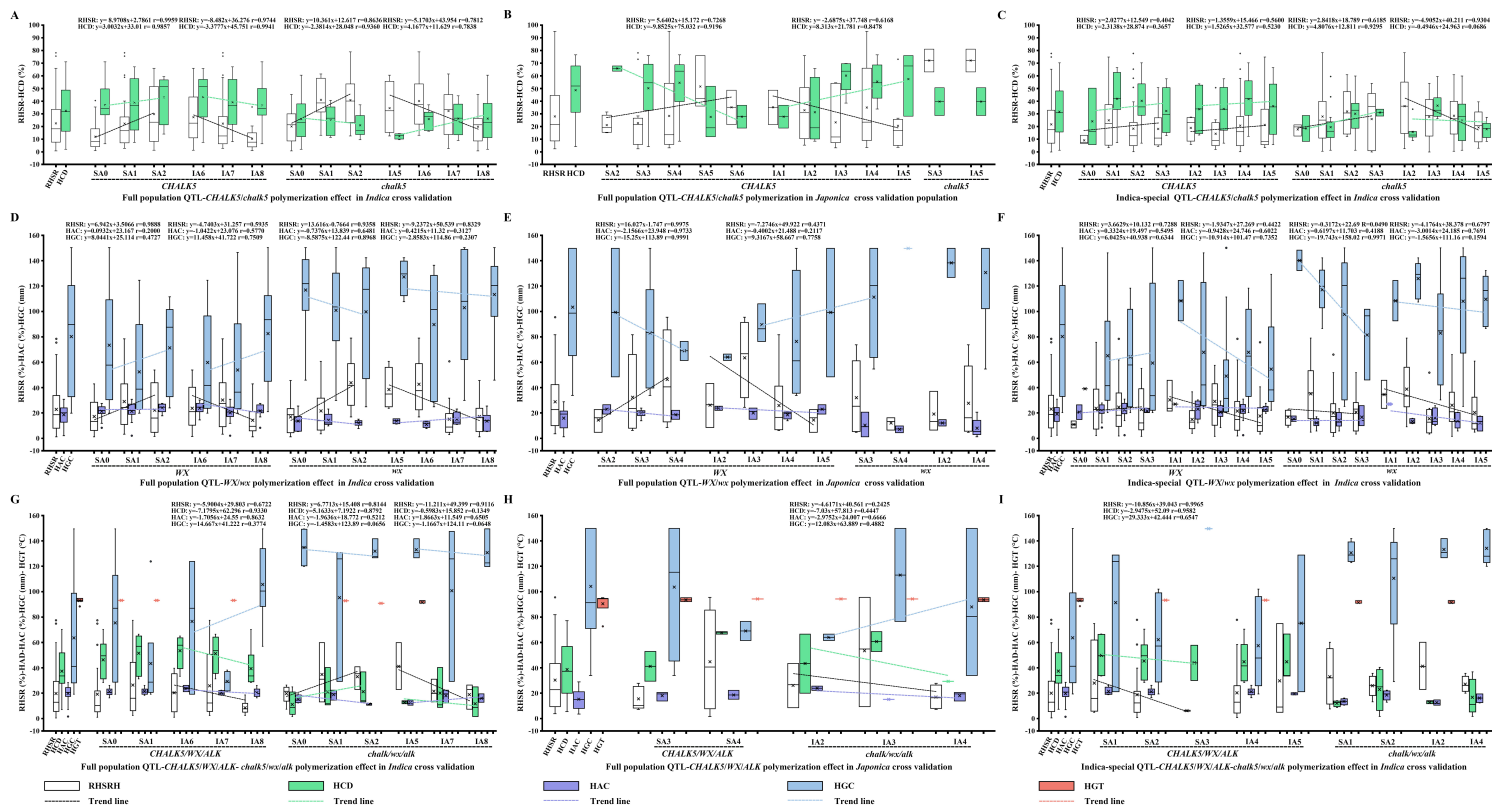

Supplement: Supplementary file 3 — Supplementary Material 3 [file 12870_2023_4260_MOESM3_ESM.pdf]

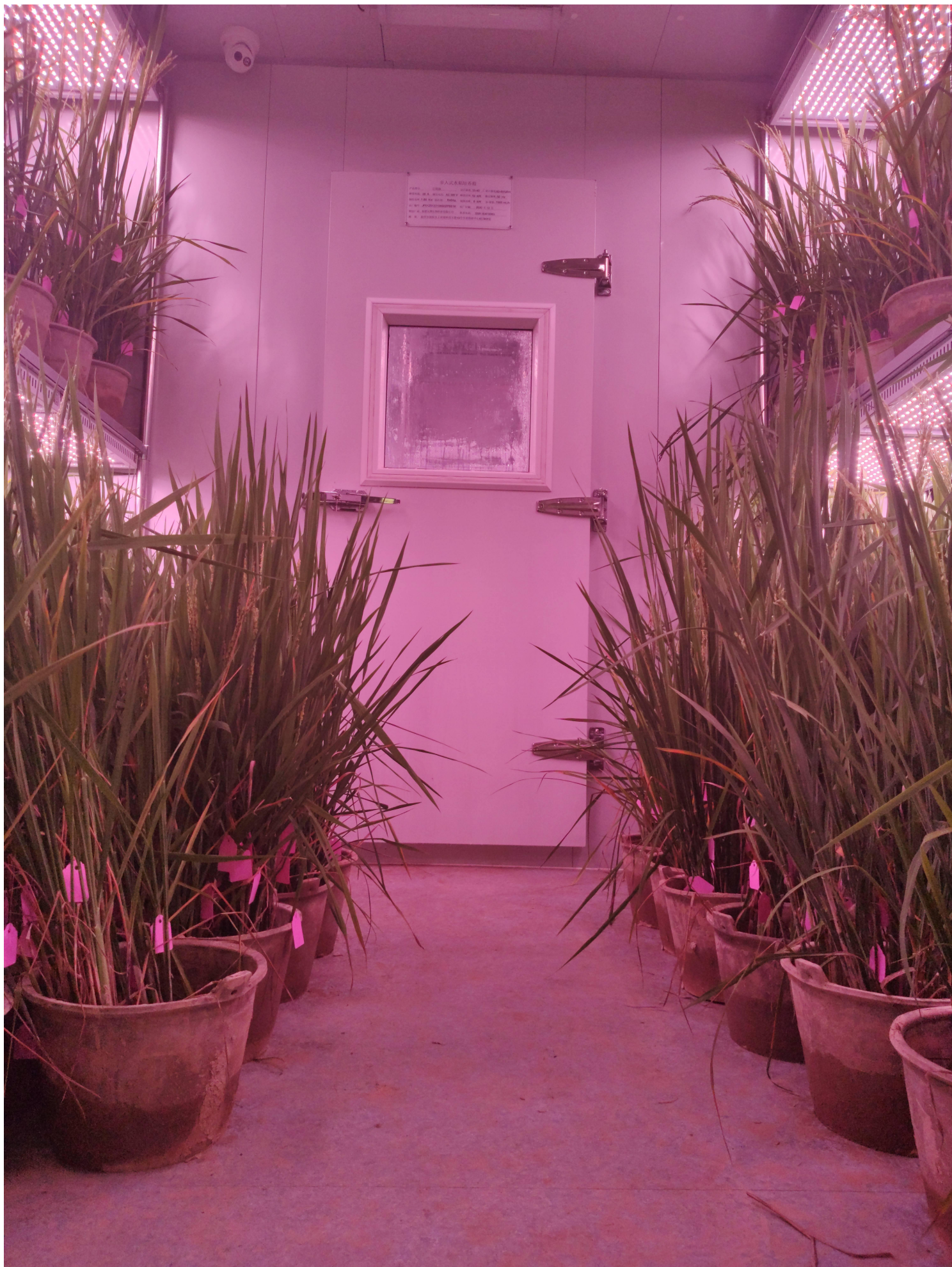

Supplement: Supplementary file 4 — Supplementary Material 4 [file 12870_2023_4260_MOESM4_ESM.pdf]
