# Supplementary material for: Utilization of natural alleles for heat adaptability QTLs at the flowering stage in rice
Source: BMC Plant Biol. 2023 May 16;23:256. doi: 10.1186/s12870-023-04260-5 (PMC10186738; doi:10.1186/s12870-023-04260-5)
Supplement: Supplementary file 5 — Supplementary Material 5 [file 12870_2023_4260_MOESM5_ESM.pdf]

Table S1 Geographic information of plant materials

No.	Population	Name	Region	Country
1	*Indica*	Jin 23B	Hunan	China
2	*Indica*	Zhong 9B	Zhejiang	China
3	*Indica*	Nan Xiong Zao You Zhan	Guangdong	China
4	*Indica*	Jia Yu 948	Zhejiang	China
5	*Indica*	Ai Tuo Gu 151	Sichuan	China
6	*Japonica*	A Er Ji Tuo	Bulgaria	Bulgaria
7	*Indica*	IR 10179-23-1-3	Philippines	Philippines
8	*Japonica*	Mu Xi Qiu	Shanghai	China
9	*Indica*	Zao Shu Xiang Hei	Guangxi	China
10	*Japonica*	Nipponbare	Japan	Japan
11	*Indica*	Zhen Shan 97B	Zhejiang	China
12	*Indica*	R287	Hubei	China
13	*Indica*	Zhong Jian 99-38	Hunan	China
14	*Japonica*	Ma Gu Zi	Shaanxi	China
15	*Indica*	Yun Hui72	Yunnan	China
16	*Japonica*	Zao Sheng Bai	Japan	Japan
17	*Japonica*	Qiu Guang Teng Xi 104 Hao	Japan	Japan
18	*Indica*	R402	Hunan	China
19	*Indica*	Xiang Zao Xian 45 Hao	Hunan	China
20	*Indica*	IR 65600-27-1-2-2	Philippines	Philippines
21	*Indica*	San Tian Nuo	Japan	Japan
22	*Indica*	Ai Jiao Nan Te	Guangdong	China
23	*Japonica*	Lao Guang Tou 83	Heilongjiang	China
24	*Japonica*	Wei Guo	Liaoning	China
25	*Indica*	Zao Xian 14	Hunan	China
26	*Indica*	T0974	Henan	China
27	*Indica*	Zr02	Hunan	China
28	*Indica*	Bo B	Guangxi	China
29	*Indica*	Sri Raja	Malaysia	Malaysia
30	*Indica*	T0463	Henan	China
31	*Indica*	Zhong You Zao 81	Hunan	China
32	*Indica*	Xie Qing Zao B	Anhui	China
33	*Indica*	Lin Yi Tang Dao	Linyi	China
34	*Indica*	Ye Tuo Zai		China
35	*Japonica*	Irat109	Brazil	Brazil
36	*Indica*	R458	Hainan	China
37	*Japonica*	Hu Hui 628	Hunan	China
38	*Indica*	Xiao Hong Gu	Yunnan	China
39	*Indica*	Chuan Xiang 29B	Sichuan	China
40	*Indica*	Jie Fang Xian	Jiangxi	China
41	*Indica*	117		China
42	*Japonica*	Gao Yang Dian Dao Da Hong Mang	Hebei	China
43	*Japonica*	La Mu Jia	Yunnan	China
44	*Japonica*	Hong Qi 5 Hao	Hunan	China
45	*Indica*	IR 8192-200-3-3-1-1	Philippines	Philippines
46	*Indica*	Xiang Hui 299	Hunan	China
47	*Indica*	Xian Luo Si Chi	Thailand	Thailand
48	*Japonica*	Irat 266	Ivory Coast	Cote d'Ivoire
49	*Japonica*	80A90Yr72078-25	Australia	Australia

50	*Indica*	Ai He Chi	Jiangxi	China
51	*Indica*	Wan Li Xian	Hunan	China
52	*Indica*	Gajale	Nepal	Nepal
53	*Indica*	2004	Guangdong	China
54	*Indica*	Liu Ye Zhan	Hubei	China
55	*Indica*	Zi Mi	Yunnan	China
56	*Indica*	IR 2061-522-6-9	Philippines	Philippines
57	*Indica*	1892S	Anhui	China
58	*Indica*	You Zhan 8 Hao	Guangxi	China
59	*Indica*	Guang Lu Ai 15-1	Guangxi	China
60	*Indica*	Hei Mi Chan	Taiwan	China
61	*Indica*	P59279		
62	*Indica*	Bala	India	India
63	*Indica*	Ming Hui 77	Fujian	China
64	*Indica*	Hong Hui 98	Anhui	China
65	*Indica*	R432	Hunan	China
66	*Indica*	Nan Jing 11 Hao	Jiangsu	China
67	*Japonica*	Yi Zhi Xiang	Fujian	China
68	*Indica*	Huan Yang Zhan	Guizhou	China
69	*Indica*	Upr 191-66	India	india
70	*Indica*	Caozhao-2		China
71	*Indica*	Iet1444	India	India
72	*Indica*	IR 68897B	Philippines	Philippines
73	*Indica*	Xian Hui 207	Hunan	China
74	*Indica*	Gui Hua Zhan	Guangxi	China
75	*Indica*	Wu Ke Zhan	Fujian	China
76	*Indica*	Inga	Australia	Australia
77	*Indica*	Ma Wei Zhan	Guizhou	China
78	*Indica*	Heng Xian Liang Chun Ben Gu	Guangxi	China
79	*Indica*	Guang Lu Ai 4 Hao	Guangdong	China
80	*Indica*	Ai Zi	Anhui	China
81	*Indica*	Xiang Wan Xian 11 Hao	Hunan	China
82	*Japonica*	Nanoay P.A	Argentina	Argentina
83	*Indica*	71011	Australia	Australia
84	*Indica*	Yue Xiang Zhan	Guangdong	China
85	*Indica*	Tkm9	India	India
86	*Indica*	IR 66897B	Philippines	Philippines
87	*Indica*	IR 58821-23-1-3-1	Philippines	Philippines
88	*Indica*	Lü Han 1 Hao	Anhui	China
89	*Indica*	Hr15	Guangdong	China
90	*Indica*	Hei Du 4	Guangdong	China
91	*Indica*	San Ke Cun	Sichuan	China
92	*Indica*	Irat144	Indonesia	Indonesia
93	*Indica*	Duo 57	Sichuan	China
94	*Indica*	Mo Li Zhan Xuan	Guangdong	China
95	*Indica*	He Mei Zhan	Guangdong	China
96	*Indica*	Ye Si Zhan	Guangdong	China
97	*Indica*	Si Li Lan Ka 1 Hao	Sri Lanka	Sri lanka
98	*Indica*	Chanh 148	Vietnam	Vietnam
99	*Japonica*	Ben Bang Gu	Yunnan	China
100	*Indica*	Ai Zi Dao	Anhui	China
101	*Indica*	Lu Hui 17	Sichuan	China
102	*Indica*	Jia Fu Zhan	Fujian	China

103	*Indica*	E Si Niu	Guangdong	China
104	*Indica*	Cs94	Vietnam	Vietnam
105	*Indica*	Guang122	Guangdong	China
106	*Indica*	Yue Tai B	Guangdong	China
107	*Indica*	Xiang Wan Xian 13 Hao	Hunan	China
108	*Indica*	Guang Hui 998	Guangdong	China
109	*Indica*	Mi Yang 46	Zhejiang	China
110	*Japonica*	Jijucas Claro	Brazil	Brazil
111	*Indica*	Ai Ma Kang	Sichuan	China
112	*Indica*	Te Qing	Guangdong	China
113	*Indica*	Q5	Vietnam	Vietnam
114	*Indica*	Huang Hua Zhan	Guangdong	China
115	*Indica*	Ce 258	Guangxi	China
116	*Indica*	Gui 362 (Guang Shi Hui Fu Xi)	Guangxi	China
117	*Indica*	R106	Hubei	China
118	*Indica*	Yzx1		
119	*Indica*	Feng Hua Zhan	Guangdong	China
120	*Indica*	Qi Li Si Miao	Guangdong	China
121	*Indica*	Yu Xiang You Zhan	Guangdong	China
122	*Indica*	Lu Hui 17	Sichuan	China
123	*Indica*	Gui 99	Guangxi	China
124	*Indica*	J34	Madagascar	Madagascar
125	*Indica*	Fl478	Philippines	Philippines
126	*Indica*	Fu 838	Sichuan	China
127	*Japonica*	Iac 150/76	Brazil	Brazil
128		Ba Wang Bian 1	Hubei	China
129	*Indica*	Xu Gu Nuo	Hunan	China
130	*Indica*	R644	Hunan	China
131	*Indica*	Om1706	Vietnam	Vietnam
132	*Indica*	Liu Sha You Zhan 2 Hao	Guangxi	China
133	*Indica*	IR 55419-04	Philippines	Philippines
134	*Indica*	611 (Quan Yin Yin Jin Er Xi Hui)	Hunan	China
135	*Indica*	Yue Jing Si Miao 2 Hao	Guangdong	China
136	*Indica*	Ce 253	Guangxi	China
137	*Indica*	K 24	Uganda	Uganda
138	*Indica*	Feng Ai Zhan	Guangdong	China
139	*Indica*	Hua Jing Xian 74	Guangdong	China
140	*Indica*	C70	Vietnam	Vietnam
141	*Indica*	IR bb60	Philippines	Philippines
142	*Indica*	Zhen Gui Ai 1 Hao	Guangdong	China
143	*Indica*	Gui Nong Zhan	Guangdong	China
144	*Japonica*	Mei Guo Huang Ke Dao	USA	USA
145	*Indica*	Bi Wu Sheng	Yunnan	China
146	*Indica*	Gu Mei 2 Hao	Sichuan	China
147	*Indica*	Peng Shan Tie Gan Zhan	Sichuan	China
148	*Indica*	Tai-Zhong-Xian 10	Taiwan	China
149	*Indica*	Chipda	India	India
150	*Indica*	Zale	Myanmar	Myanmar
151	*Indica*	Gui 649	Guangxi	China
152	*Indica*	Arrozvermelho	Philippines	Philippines
153	*Indica*	Hong Wan 1 Hao	Fujian	China
154	*Indica*	Hua 565	Hubei	China
155	*Indica*	Hu Han 15	Shanghai	China

156	*Indica*	Quan Zhen 10 Hao	Fujian	China
157	*Indica*	Shu Hui 527	Sichuan	China
158	*Indica*	Zhong Guang Xiang 1 Hao	Guangxi	China
159	*Indica*	Han Hui 10 Hao	Shanghai	China
160	*Indica*	Shu Hui 498	Sichuan	China
161	*Indica*	Shu Hui 527	Sichuan	China
162	*Indica*	D11		China
163	*Indica*	Kcd1		
164	*Japonica*	Ita 221	Nigeria	Nigeria
165	*Indica*	Chhomromg	Nepal	Nepal
166	*Indica*	Palung 2	Nepal	Nepal
167	*Indica*	Tek Si Chut	Taiwan	China
168	*Indica*	Kasalath	Japan	Japan
169	*Indica*	Ir64-Il		China
170	*Indica*	Bw311-9	Philippines	Philippines
171	*Indica*	Sagc－4		China
172	*Indica*	Wan Hui 057	Anhui	China
173	*Indica*	R9308	Zhejiang	China
174	*Indica*	Huang Si Gui Zhan	Guangdong	China
175	*Indica*	At354	Sri Lanka	Sri Lanka
176	*Indica*	Tn1	Taiwan	China
177	*Indica*	Gui Hui 5832	Guizhou	China
178	*Indica*	G341	Hungary	Hungary
179	*Indica*	Yi Hui 1577	Sichuan	China
180		Ji Nei Ya Dao	Guinea	Guinea
181	*Indica*	Br11	Bangladesh	Bangladesh
182	*Indica*	Zi Hui 100	Anhui	China
183	*Indica*	Ir68552-55-3-2	Philippines	Philippines
184	*Indica*	IR bb7	Philippines	Philippines
185	*Indica*	Ming Hui 86	Fujian	China
186	*Indica*	Zhe Hui 7954	Zhejiang	China
187	*Indica*	B6136-3-Tb-0-1-5	Philippines	Philippines
188	*Indica*	Zhong Hui 8006	Zhejiang	China
189	*Indica*	Hao Lai	Yunnan	China
190	*Indica*	Hnankar	Myanmar	Myanmar
191	*Indica*	Zhong Hui 8006	Zhejiang	China
192	*Indica*	En Hui 58	Hubei	China
193	*Indica*	Bao Xuan 21 Hao	Guangdong	China
194	*Indica*	Irbb62	Philippines	Philippines
195	*Indica*	Cheng Hui 727	Sichuan	China
196	*Indica*	Yue Hui 9113	Hunan	China
197	*Indica*	Zhe Hui 7954	Zhejiang	China
198	*Indica*	Cheng Hui 448	Sichuan	China
199	*Indica*	Zhong 413	Zhejiang	China
200	*Indica*	Jiang Xi Si Miao	Jiangxi	China
201	*Indica*	9516	Zhejiang	China
202	*Indica*	D15		China
203	*Indica*	Gan Wan Xian 37 Hao（926)	Jiangxi	China
204	*Indica*	Xian Xiao Zhan	Guangdong	China
205	*Indica*	108S	Liaoning	China
206	*Indica*	Zhong Hua 1 Hao	Guangdong	China
207	*Indica*	Nerica-L-1		Africa
208	*Indica*	1088		

209	*Indica*	Ming Hui 63	Fujian	China
210	*Indica*	84	Jiangsu	China
211	*Indica*	Cdr22	Sichuan	China
212	*Indica*	Pr106	India	India
213	*Indica*	Irat 352	Colombia	Columbia
214	*Indica*	IR 71466-75-3-B-1	Philippines	Philippines
215	*Indica*	IR 24	Philippines	Philippines
216	*Indica*	Slg1	Japan	Japan
217	*Indica*	Zhong Hui 8015	Zhejiang	China
218	*Indica*	84	Jiangsu	China
219	*Indica*	IR 64-Il		China
220	*Indica*	Gui Hui 2190	Guizhou	China
221	*Indica*	Hua 564	Hubei	China
222	*Indica*	Psbrc82	Philippines	Philippines
223	*Indica*	Kr200	Thailand	Thailand
224	*Indica*	Duo Xi 1 Hao	Sichuan	China
225	*Indica*	Ming Hui 86	Fujian	China
226	*Indica*	Min Hui 3301	Fujian	China
227	*Indica*	Wh26	Anhui	China
228	*Indica*	Cdr22	Sichuan	China
229	*Indica*	Cheng Hui 177	Sichuan	China
230	*Indica*	Dang Yu 5 Hao	Anhui	China
231	*Indica*	Hong Jing Han Gu	Guangxi	China
232	*Indica*	3027	Zhejiang	China
233	*Indica*	Hei He Ai Hui	Heilongjiang	China
234	*Indica*	Nerica-L-27		Africa
235	*Indica*	Adny 11		Africa
236	*Indica*	Mian Hui 725	Sichuan	China
237	*Indica*	Ir58025B	Philippines	Philippines
238	*Indica*	Ir64	Philippines	Philippines
239	*Indica*	Basmati	India	India
240	*Indica*	Ir64-Il		China
241	*Indica*	Meng Guan Da Ma Gu	Guizhou	China
242	*Indica*	Wan Xian 77	Anhui	China
243	*Indica*	Psbrc80	Philippines	Philippines
244	*Indica*	Hang 1 Hao	Fujian	China
245	*Indica*	Yang Dao 2 Hao	Jiangsu	China
246	*Indica*	Dian Tun 502	Yunnan	China
247	*Indica*	Chorofa	Philippines	Philippines
248	*Indica*	IR 64A	Philippines	Philippines
249	*Indica*	Brc 25-146-2-1	Bangladesh	Bangladesh
250	*Indica*	Da Wan Nuo	Yunnan	China
251	*Indica*	Mo Mi	Guangxi	China
252	*Indica*	IR 50	Philippines	Philippines
253	*Indica*	X23	Vietnam	Vietnam
254	*Indica*	Ajaya	India	india
255	*Indica*	Budda	India	india
256	*Indica*	X22	Vietnam	Vietnam
257	*Indica*	Gz 1368-5-4	Egypt	Egypt
258	*Indica*	Gui Hui 168	Guizhou	China
259	*Indica*	Matatag2	Philippines	Philippines
260	*Indica*	Yan Hui 559	Jiangsu	China
261	*Indica*	Rohini	India	India

262	*Indica*	Jwr 221	Jiangsu	China
263	*Indica*	Ba Bao Mi	Yunnan	China
264	*Indica*	IR6	Pakistan	Pakistan
265	*Indica*	X21	Vietnam	Vietnam
266	*Indica*	Nonabokra	India	India
267	*Indica*	Lx2007		China
268	*Indica*	B5-10	Hubei	China
269	*Indica*	Nionoka	Mali	Mali
270	*Indica*	Ming Hui 63	Fujian	China
271	*Indica*	9311	Jiangsu	China
272	*Indica*	Gao Zi		China
273	*Indica*	IR 06G113	Philippines	Philippines
274	*Indica*	IR 74	Philippines	Philippines
275	*Indica*	Zh5	Zhejiang	China
276	*Indica*	B5	Hubei	China
277	*Indica*	Bg94-1	Sri Lanka	Sri Lanka
278	*Indica*	Govind	India	india
279	*Indica*	Rnr 67580	India	India
280	*Indica*	Pms 10B	India	India
281	*Indica*	IR 52561-Ubn-1-1-2	Philippines	Philippines
282	*Indica*	Nsic Rc9 (Apo)	Philippines	Philippines
283	*Indica*	Npt-114	Philippines	Philippines
284	*Indica*	Te Xian Zhan 25	Guangdong	China
285	*Indica*	Gan Wan Xian 30 Hao	Jiangxi	China
286	*Indica*	2037 (Rajahamsal)	India	India
287	*Indica*	C 894-21	Philippines	Philippines
288	*Indica*	Er Gang Ai	Guangdong	China
289	*Indica*	Bg90-2	Sri Lanka	Sri Lanka
290	*Indica*	Hui 752	Jiangxi	China
291	*Indica*	Sard		
292	*Indica*	Cisanggarung	Indonesia	Indonesia
293	*Indica*	C.Medio 7	Cuba	Cuba
294	*Indica*	Tun Sart	Vietnam	Vietnam
295	*Indica*	Kogomg 1-1		
296	*Indica*	33 (Quan Yin Yin Jin Er Xi Hui)	Hubei	China
297	*Indica*	Cisadane	Indonesia	Indonesia
298	*Indica*	IR 42	Philippines	Philippines
299	*Indica*	Ngatsin	India	India
300	*Japonica*	Wu Zi Luo Si 215	Russia	Russia
301	*Japonica*	Huang Pi Nuo	Yunnan	China
302	*Indica*	Ning Hui 21	Jiangsu	China
303	*Indica*	Y134	Anhui	China
304	*Indica*	Le Hui 188	Sichuan	China
305	*Japonica*	Jiang Hua Dao	North Korea	North Korea
306	*Japonica*	Qing Jin Zao Sheng	North Korea	North Korea
307	*Japonica*	Hei Biao	North Korea	North Korea
308	*Japonica*	San Sui Jin	Japan	Japan
309	*Japonica*	Zhu Yuan	Japan	Japan
310	*Japonica*	Wan Shi	Japan	Japan
311	*Japonica*	Ai Lu Yu	Japan	Japan
312	*Indica*	Ai Yi Si	Vietnam	Vietnam
313	*Indica*	Tian Han Dao	Vietnam	Vietnam
314	*Indica*	Yue Nan Zao Dao	Vietnam	Vietnam

315	*Indica*	Co 22	India	India
316	*Japonica*	Hong Se 90	Russia	Russia
317	*Indica*	Ka Ha Mu	Romanian	Romania
318	*Japonica*	Ao Mi Er Te 168	Hungary	Hungary
319	*Japonica*	A Er Ba Ni Ya	Albania	Albania
320	*Japonica*	Lin Guo	Italy	Italy
321	*Indica*	Bu Lei Da A-75	Mexico	Mexico
322	*Japonica*	Khao Mack Kheua	Laos	Laos
323		Tjantajan	Indonesia	Indonesia
324	*Indica*	IR 50	Philippines	Philippines
325	*Indica*	Basmati 443	Pakistan	Pakistan
326	*Japonica*	Nabated A Smar	Egypt	Egypt
327	*Indica*	Pelde	Australia	Australia
328	*Japonica*	Yr 83-23-11	Australia	Australia
329	*Japonica*	Chi Mao	Japan	Japan
330	*Japonica*	Gong Cheng Xiang	Japan	Japan
331		Zhen Fu 8	Japan	Japan
332	*Japonica*	Qing Nuo キョハタモチ	Japan	Japan
333	*Indica*	Cisokan	Indonesia	Indonesia
334	*Aus*	Za Cao Dao 13	Nepal	Nepal
335	*Indica*	Br 2029-2-2-2	Bangladesh	Bangladesh
336	*Indica*	Jhona 349	India	India
337	*Indica*	Rp 1570-44-1	India	India
338	*Japonica*	Yr196	Australia	Australia
339	*Indica*	Ecia 179-S13	Cuba	Cuba
340	*Japonica*	Tie Gan Wu	Wuxing County	China
341	*Indica*	Er Jiu Nan 1 Hao	Jiaxing area	China
342	*Indica*	Guang Lu Ai 4 Hao	Guangzhou	China
343	*Indica*	Gui Chao 2 Hao	Guangzhou	China
344	*Japonica*	Tai Dong Lu Dao 328	Taiwan	China
345	*Indica*	Tai Zhong Xian Xuan 220 Hao	Taiwan	China
346	*Indica*	Xiang Zao Xian 7 Hao	Huaihua area	China
347	*Indica*	Cheng Nong Shui Jing Mi	Chengdu	China
348	*Japonica*	Guang Ke Xiang Nuo	Fengshan	China
349	*Indica*	Fu Ning Zi Pi Jing Zi	Funing County	China
350	*Japonica*	Long Hua Mao Hu Lu	Longhua County	China
351	*Japonica*	Bai Mao Dao	Suihua County	China
352	*Japonica*	You Mang Zao Jing	Fengxian County	China
353	*Japonica*	Cun San Li	Nantong County	China
354	*Indica*	Qiu Qian Bai	Huaining County	China
355	*Japonica*	Fei Dong Tang Dao	Feidong County	China
356	*Indica*	Jin Xi Bai	Dongxiang County	China
357	*Indica*	Min Bei Wan Xian	Jianning County	China
358	*Indica*	Lu Cai Hao	Xianyou County	China
359	*Indica*	Shu Ya Zhan	Dianbai County	China
360	*Indica*	Qi Mei	Yingde County	China
361	*Indica*	Bai Ke Hua Luo	Guangdong	China
362	*Japonica*	Chi Ke Nuo	Guangdong	China
363	*Indica*	Qi Yue Xian	Du'an County	China
364	*Indica*	Dong Ting Wan Xian	Chongyang County	China
365	*Indica*	Xuan En Chang Tan Qing Zhan	Xuan'en County	China
366	*Indica*	Han Ma Dao	Huaibin County	China
367	*Japonica*	Xi Bai Zhan	Mianning County	China

368	*Indica*	Zhong Nong 4 Hao	Wanxian area	China
369	*Indica*	Hong Gu	Nanjiang County	China
370	*Indica*	Qi Tou Bai Gu	Lincang County	China
371	*Indica*	Gong Ju 73	Menglian County	China
372	*Indica*	Zi Nuo	Pu'er County	China
373	*Indica*	Jin Zhi Nuo	Cloud County	China
374	*Indica*	Ji Xie Nuo	Gengma County	China
375	*Indica*	Wu Ju Hong Gu	Xinping County	China
376	*Japonica*	Ze Gu	Zunyi County	China
377	*Indica*	Xiang Nuo	Zhenfeng County	China
378	*Indica*	Zhan Ke Nuo	Fuquan County	China
379	*Japonica*	Hong Ke Zhe Nuo (2)	Jianhe County	China
380	*Japonica*	Cun Gu Nuo	Wuchuan County	China
381	*Japonica*	Guan Tui Bai He 1	Congjiang County	China
382	*Japonica*	Hei Mang Dao	Zhongwei County	China
383	*Japonica*	Pu Tao Huang	Baodi County	China
384	*Japonica*	Hao Ba Yong 1	Menglian County	China
385	*Japonica*	Leng Shui Gu 2	Lancang County	China
386	*Indica*	Men Jia Ding 2	Ledong County	China
387	*Japonica*	Ba Bai Li	Longling County	China
388	*Indica*	Liu Sha 1 Hao	Liuzhou area	China
389	*Indica*	Chen Wan 3 Hao	Chenzhou area	China
390	*Japonica*	Li Xin Jing	Xichang area	China
391	*Indica*	Xiang Ai Zao 10 Hao	Changsha	China
392	*Indica*	Lu Ke 3 Hao	Chengdu	China
393	*Japonica*	Liao Jing 287	Shenyang	China
394	*Indica*	Zao Xian 240	Xuancheng area	China
395	*Indica*	Ai Zai Zhan	Teng County	China
396	*Indica*	80B	Hunan	China
397	*Indica*	Gui 630	Hunan	China
398	*Japonica*	Jing 7623	Shanghai	China
399	*Japonica*	Hao Lü Guang Zhan	Ceheng County	China
400	*Indica*	L301B	Hunan	China
401		Zao Shu Nong Hu 6 Hao B	Hunan	China
402	*Indica*	Qing Si Ai 16B	Guangdong	China
403	*Indica*	Xian Gai B	Jiangxi	China
404	*Japonica*	Li Ming B	Liaoning	China
405	*Indica*	Bao Xie -7B	Hunan	China
406	*Indica*	88B	Jiangsu	China
407	*Japonica*	Xing Guo	Huaide County	China
408	*Indica*	Lei Huo Zhan	Taihu County	China
409		Tai Zhong 65 Hao / Tai Zhong Hr539	Taiwan	China
410	*Indica*	Tai Zhong Zai Lai 1 Hao/Tai Zhong 6[illegible]	Taiwan	China
411	*Indica*	Ma Ma Gu	Gulin County	China
412	*Japonica*	Ye Li Cang Hua	Tianjin	China
413	*Japonica*	Huang Ke Zao Nian Ri	Wuxi County	China
414	*Japonica*	Bai Ge Dao	Jiangyin County	China
415	*Indica*	Liu Shi Zao	Wuhu county	China
416	*Indica*	San Bai Li	Jiangxi	China
417	*Japonica*	Hao Ma Ke (K)	Menglian County	China
418	*Indica*	9311	Jiangsu	China
419	*Japonica*	C418	Liaoning	China
420	*Japonica*	Yun Jing 7	Yunnan	China

421	*Japonica*	Milyang23	North Korea	North Korea
422		Shwe Thwe Yin Hyv	Myanmar	Myanmar
423		Bg300	Sri Lanka	Sri Lanka
424	*Aus*	Om997	Vietnam	Vietnam
425		Psbrc28		
426		Psbrc66		
427	*Japonica*	Yun Guang 8 Hao	Yunnan	China
428	*Japonica*	Zhong Chao 123	Zhejiang	China
429	*Japonica*	Giza159	Egypt	Egypt
430		Jhona 349	India	India
431	*Japonica*	Domsiah	Iran	Iran
432		Jp-5	Pakistan	Pakistan
433		M202	USA	USA
434	*Aro*	Up15	Japan	Japan
435	*Japonica*	Wu Da Dao Zhong	North Korea	North Korea
436	*Japonica*	Qd_441		
437	*Japonica*	Amareles	Portugal	Portugal
438	*Japonica*	Ai Yeh Lu		
439	*Japonica*	Azucena	Filipin	Filipin
440	*Aro*	Tetep	India	India
441	*Japonica*	Wu Yu Jing 3 Hao	Jiangsu	China
442	*Japonica*	Wu Yu Jing 14 Hao	Jiangsu	China
443	*Japonica*	Chang Bai 9 Hao	Jilin	China
444	*Japonica*	Hua Yu 560	Tianjin	China
445		Pokhreli		
446	*Japonica*	Song Jing 5 Hao	Heilongjiang	China
447	*Japonica*	Liao Xing 1 Hao	Liaoning	China
448		Qd_471		
449	*Japonica*	Nong Ken 58	Japan	Japan
450	*Japonica*	Shen Nong 89366	Liaoning	China
451		Ba Xi Nuo Dao	Brazil	Brazil
452		F6		
453	*Japonica*	Irat109	Brazil	Brazil
454		Ct9993-5-10-1-M		
455		IR 55411-53	Philippines	Philippines
456	*Japonica*	Han 277	Beijing	China
457	*Japonica*	Y16B	Jilin	China
458		Padisenemok		
459	*Japonica*	Dinorado	Philippines	Philippines
460	*Japonica*	02428-Il	Jiangsu	China
461		Maravilha	Brazil	Brazil
462		Li Jiang Xin Tuan Hei Gu	Yunnan	China
463		Chang Ye Bao Chi Xi	Japan	Japan
464		C349		
465		Chao Hui 一1	Zhejiang	China
466		Sb90		
467	*Japonica*	Wu Yun Jing 7 Hao	Jiangsu	China
468	*Japonica*	Zi Dao	Yunnan	China
469	*Japonica*	Mr39	Anhui	China
470	*Japonica*	Mr19	Anhui	China
471	*Japonica*	Zhen Dao 88	Jiangsu	China
472	*Japonica*	Nan Jing 45	Jiangsu	China
473		Yue Guang	Japan	Japan

474	*Japonica*	Song 820	Heilongjiang	China
475	*Japonica*	Xu Dao 3 Hao	Jiangsu	China
476	*Japonica*	Huai Dao 9 Hao	Jiangsu	China
477		Hao Ge Lao	Yunnan	China
478	*Japonica*	Hong Za		
479	*Japonica*	Feng Za		
480	*Japonica*	Guan Dong 194 (Ruan Mi)	Japan	Japan
481	*Japonica*	Yang Jing 4038	Jiangsu	China
482	*Japonica*	Zhen Dao 99	Jiangsu	China
483	*Japonica*	Wu Jing 15	Jiangsu	China
484	*Japonica*	Wu Yun Jing 21	Jiangsu	China
485	*Japonica*	Hua Jing 6 Hao	Jiangsu	China
486	*Japonica*	Nan Jing 44	Jiangsu	China
487	*Japonica*	Wu Xiang Jing 14 Hao	Jiangsu	China
488	*Japonica*	Xu Dao 5 Hao	Jiangsu	China
489	*Japonica*	Lian Jing 4 Hao	Jiangsu	China
490	*Japonica*	Chang Nong Jing 5 Hao	Jiangsu	China
491	*Japonica*	Wu Yun Jing 19 Hao	Jiangsu	China
492	*Japonica*	Ken Dao 12	Heilongjiang	China
493	*Japonica*	Ken Jian Dao 6	Heilongjiang	China
494	*Japonica*	Long Dao 5	Heilongjiang	China
495	*Japonica*	Long Jing 20	Heilongjiang	China
496	*Japonica*	Long Jing 21	Heilongjiang	China
497	*Japonica*	Long Jing 24	Heilongjiang	China
498	*Japonica*	Long Jing 26	Heilongjiang	China
499	*Japonica*	San Jiang 1	Heilongjiang	China
500	*Japonica*	Song 01-173	Heilongjiang	China
501	*Japonica*	Sui Jing 4	Heilongjiang	China
502	*Japonica*	Sui Jing 7	Heilongjiang	China
503	*Japonica*	Sui Jing 8	Heilongjiang	China
504	*Japonica*	Sui Jing 9	Heilongjiang	China
505	*Japonica*	Tong Zhan 1 Hao	Jilin	China
506	*Japonica*	Xiu Shui 123	Zhejiang	China
507	*Japonica*	Xiu Shui 128	Zhejiang	China
508	*Japonica*	Jia 991	Zhejiang	China
509	*Japonica*	Lian Jing 6 Hao	Jiangsu	China
510	*Japonica*	Jin Zao 47	Zhejiang	China
511	*Japonica*	Liao Jing 294	Liaoning	China
512	*Japonica*	Liao Jing 454	Liaoning	China
513	*Indica*	Wantai B	Nanning	China
514	*Indica*	Gui117	Nanning	China
515	*Indica*	Gui726	Nanning	China
516	*Indica*	Gui298	Nanning	China
517	*Indica*	Guiyefeng	Nanning	China
