# Supplementary material for: Utilization of natural alleles for heat adaptability QTLs at the flowering stage in rice
Source: BMC Plant Biol. 2023 May 16;23:256. doi: 10.1186/s12870-023-04260-5 (PMC10186738; doi:10.1186/s12870-023-04260-5)
Supplement: Supplementary file 6 — Supplementary Material 6 [file 12870_2023_4260_MOESM6_ESM.pdf]

Table S2 Information of strong heat-tolerant cultivars

**No.**	**Name**	**Subspecies**	**Origin**	**Type**	**SSR (%)**	**HSR (%)**	**RHSR (%)**
1	Jia Yu 948	*Indica*	China	Improved variety	70.17	53.90	76.81
2	IR 65600-27-1-2-2	*Indica*	Philippines	Introduced variety	77.62	68.37	88.08
3	Ye Tuo Zai	*Indica*	China	Landrace	75.15	56.16	74.74
4	Zi Mi	*Indica*	China	Landrace	70.47	58.92	83.62
5	Hei Mi Chan	*Indica*	China	Landrace	72.18	67.58	93.63
6	Inga	*Indica*	Australia	Introduced variety	74.07	57.95	78.24
7	Qi Li Si Miao	*Indica*	China	Improved variety	79.33	65.53	82.60
8	Yue Jing Si Miao 2 Hao	*Indica*	China	Improved variety	74.39	57.74	77.61
9	Ken Jian Dao 6	*Japonica*	China	Improved variety	90.50	63.43	70.10
10	Long Jing 26	*Japonica*	China	Improved variety	86.92	64.19	73.86
11	Sui Jing 7	*Japonica*	China	Improved variety	76.12	55.09	72.38
12	Liao Jing 294	*Japonica*	China	Improved variety	85.66	67.62	78.95
13	Wei Guo	Temperate *japonica*	China	Landrace	77.95	65.50	84.03
14	IRAT109	Tropical *japonica*	Brazil	Improved variety	71.65	58.59	81.78
15	Guan Dong 194	Temperate *japonica*	Japan	Improved variety	86.46	64.54	74.65
16	Tong Zhan 1 Hao	Temperate *japonica*	China	Improved variety	80.64	68.53	84.98
