# Supplementary material for: Utilization of natural alleles for heat adaptability QTLs at the flowering stage in rice
Source: BMC Plant Biol. 2023 May 16;23:256. doi: 10.1186/s12870-023-04260-5 (PMC10186738; doi:10.1186/s12870-023-04260-5)
Supplement: Supplementary file 7 — Supplementary Material 7 [file 12870_2023_4260_MOESM7_ESM.pdf]

Table S3 Chalkiness degree of QTL and *CHALK5* /*chalk5* under normal condition and heat stress in *indica*

**Genotype**	**Normal condition**	**Heat stress**
qHTT1/CHALK5	21.43±14.3	35.52±16.5
qHTT3.1/CHALK5	27.71±23.25	48.66±27.52
qHTT3.2/CHALK5	27.71±23.25	48.66±27.52
qHTT4.1/CHALK5	21.72±19.61	39.21±26.87
qHTT4.2/CHALK5	25.06±16.57	40.68±20.3**
qHTT5/CHALK5	22.79±10.98	27.17±14.57
qHTT7.1/CHALK5	46.26±15.96	62.4±5.21
qHTT7.2/CHALK5	14.02±12.53	35.78±17.12
qHTT3.1- X*/CHALK5*	34.03±23.69	62.76±14.67
qHTT3.2- X*/CHALK5*	20.07±14.07	40.5±21.59
qHTT3.3- X*/CHALK5*	32.34±19.69	35.72±22.48**
qHTT4- X*/CHALK5*	25.12±15.77	44.3±19.33
qHTT5- X*/CHALK5*	25.31±20.47	44.13±24.01
qHTT12- X*/CHALK5*	22.64±14.3	38.46±24.28
qHTT1/chalk5	7.69±5.27	23.1±12.06**
qHTT4.2/chalk5	13.41±13.58	27.59±15.79**
qHTT3.2- X*/chalk5*	12.61±9.21	31.36±15.74**
qHTT4- X*/chalk5*	13.81±13.45	31.26±16.62**
qHTT2- X*/chalk5*	11.51±10.78	31.48±14.82**

Note: * means significant difference between chalkiness degree under normal condition and under heat stress, ** means highly significant difference between chalkiness degree under normal condition and under heat stress.
