# Supplementary material for: Utilization of natural alleles for heat adaptability QTLs at the flowering stage in rice
Source: BMC Plant Biol. 2023 May 16;23:256. doi: 10.1186/s12870-023-04260-5 (PMC10186738; doi:10.1186/s12870-023-04260-5)
Supplement: Supplementary file 8 — Supplementary Material 8 [file 12870_2023_4260_MOESM8_ESM.pdf]

Table S4 Multiple genotype-hap1 and phenotype

QTL	Genotype	RHSR(%)	HCD	HAC (%)	HGC (mm)	HGT (°C)
Indica	-	24.88±20.84	36.79±19.47	18.38±7.3	65.03±37.02	89.48±3.82
Japonica	-	31.37±21.41	45.4±20.16	12.76±5.09	96.13±36.1	93.14±1.47
Full QTL in *indica*	*qHTT1 /qHTT3.1 /qHTT3.2 /qHTT4.1 /qHTT5 /qHTT7.2 /CHALK5*	73.26±4.97**	24.61±18.24			
Full QTL in *indica*	*qHTT1 /qHTT4.1 /CHALK5*	48.61±18.82**	32.46±11.82			
Full QTL in *indica*	*qHTT1 /qHTT4.1 /chalk5*	49.65±25.4**	21.99±12.58*			
Full QTL in *japonica*	*qHTT1 /qHTT3.1 /qHTT3.2 /CHALK5*	16.46±14.05**	50.26±18.79			
Full QTL in *japonica*	*qHTT1 /qHTT3.1 /qHTT3.2 /qHTT4.1 /CHALK5*	35.11±6.46	34.81±24.85			
Full QTL in *japonica*	*qHTT1 /qHTT3.1 /qHTT3.2 /qHTT7.1 /CHALK5*	43.47±22.72	43.48±17.47			
Indica -specific QTL in *indica*	*qHTT3.1-X /qHTT4-X /CHALK5*	20.95±17.77	47.45±17.84**			
Indica -specific QTL in *indica*	*qHTT3.1-X /qHTT4-X /qHTT12-X /CHALK5*	36.63±14.75	41.59±24.44			
Indica -specific QTL in *indica*	*qHTT3.1-X /qHTT12-X /CHALK5*	23.86±16.25	31.28±32.1			
Indica -specific QTL in *indica*	*qHTT3.1-X /qHTT12-X /chalk5*	16.77±12.01	31.69±15.52			
Indica -specific QTL in *indica*	*qHTT3.1-X /qHTT4-X /qHTT12-X /chalk5*	27.81±19.88	29.84±14.92			
Full QTL in *indica*	*qHTT1/qHTT4.2/WX*	55.79±19.37**		26.16±2.27*	29.16±8.47*	
Full QTL in *indica*	*qHTT1/qHTT4.2/wx*	41.5±24.86**		12.46±3.53**	76.5±23.63	
Full QTL in *japonica*	*qHTT1/qHTT3.1/qHTT3.2/WX*	32.74±20.06		18.05±5.54	36.66±8.17**	
Full QTL in *japonica*	*qHTT3.1/qHTT3.2/WX*	21.89±12.99		20.75±4.14**	57.25±29.48*	
Full QTL in *japonica*	*qHTT1/qHTT3.1/qHTT3.2/wx*	13.96±9.26*		9.78±4.38	93.4±29.06	
Full QTL in *japonica*	*qHTT1/qHTT3.1/qHTT3.2/qHTT4.1/wx*	34.92±8.14		10.1±4.16	136.25±18.21*	
Full QTL in *japonica*	*qHTT1/qHTT3.1/qHTT3.2/qHTT5/wx*	22.85±4.18		7.73±1.01	100±28	
Full QTL in *japonica*	*qHTT1/qHTT3.1/qHTT3.2/qHTT7.1/wx*	40.04±21.6		12.98±2.7	118.77±19.24	
Full QTL in *japonica*	*qHTT1/qHTT3.1/qHTT3.2/qHTT4.1/qHTT7.1/qHTT7.2/wx*	51.63±18.02		13.13±0.02	107.5±28.5	
Full QTL in *japonica*	*qHTT3.1/qHTT3.2/wx*	21.2±17.74		11.8±1.07	104.6±19.35	
Full QTL in *japonica*	*qHTT3.1/qHTT3.2/qHTT4.1/wx*	27.58±13.61		12.97±1.58	91±13.44	
Full QTL in *japonica*	*qHTT3.1/qHTT3.2/qHTT7.1/wx*	26.85±6.23		11.02±1.8	88.66±24.55	
Indica -specific QTL in *indica*	*qHTT3.2-X/qHTT4.2-X/WX*	25.24±20.5		23.42±4.71**	42.53±28.55*	
Indica -specific QTL in *indica*	*qHTT3.2-X/qHTT3-X/qHTT12-X/WX*	36.3±19.28		23.96±2.66*	58.37±44.63	

Indica -specific QTL in *indica*	*qHTT3.2-X/qHTT12-X/WX*	29.22±15.3		27.63±2.73	44±20	
Indica -specific QTL in *indica*	*qHTT3.2-X/qHTT4-X/wx*	28.75±22.05		13.1±5.93**	80±35.2	
Indica -specific QTL in *indica*	*qHTT3.2-X/qHTT3-X/qHTT12-X/wx*	29.16±14.74		13.04±7.37*	80.5±31.17	
Full QTL in *indica*	*qHTT1/qHTT4.2/ALK*	53.98±21.96**				86.97±3.17
Full QTL in *indica*	*qHTT1/qHTT4.2/alk*	45.4±23.81				88.82±1.89
Indica -specific QTL in *indica*	*qHTT3.2-X/qHTT4-X/ALK*	21.94±19.22				90.26±3.57
Indica -specific QTL in *indica*	*qHTT3.2-X/qHTT4-X/qHTT12-X/alk*	28.53±15.52				87.8±3.45
Indica -specific QTL in *indica*	*qHTT3.2-X/qHTT2-X/alk*	28.97±15.57				87.42±1.12
Indica -specific QTL in *indica*	*qHTT3.2-X/qHTT4-X/alk*	32.5±21.72				88.91±4.51
Indica -specific QTL in *indica*	*qHTT3.2-X/qHTT4-X/qHTT12-X/alk*	43.69±16.26*				88.86±1.33
Full QTL in *japonica*	*qHTT1/qHTT3.1/qHTT3.2/ALK*	21.9±20.1				91.75±1.59
Full QTL in *japonica*	*qHTT1/qHTT3.1/qHTT3.2/alk*	14.62±11.4*				91.8±0.75
Full QTL in *japonica*	*qHTT1/qHTT3.1/qHTT3.2/qHTT7.1/alk*	42.51±24.77				91.75±0.79

Note: RHSR, HCD, HAC, HGC, HGT represent average relative seed setting rate under heat stress, chalkiness degree, amylose content, gel consistency, and gelatinization temperature under heat stress, respectively. * means significant difference between hap1 and hap2, ** means highly significant difference between Hap1 and Hap2.

S
