# Supplementary material for: Utilization of natural alleles for heat adaptability QTLs at the flowering stage in rice
Source: BMC Plant Biol. 2023 May 16;23:256. doi: 10.1186/s12870-023-04260-5 (PMC10186738; doi:10.1186/s12870-023-04260-5)
Supplement: Supplementary file 9 — Supplementary Material 9 [file 12870_2023_4260_MOESM9_ESM.pdf]

Table S5 RHSR, HCD, HAC, HGC, HGT of heat-tolerant QTL with *CHALK5/WX/ALK* or *chalk5/wx/alk in indica*

**Genotype**	**RHSR（%）**	**HCD**	**HAC（%）**	**HGC（mm)**	**HGT (°C)**
qHTT1 -Hap1/*CHALK5/WX/ALK*	49.77±29.91*	49.28±10.94	23.37±2.05	97.5±52.5	89.42±5.62
qHTT3.1 -Hap1/*CHALK5/WX/ALK*	81.84±10.42**	62.76±14.67	22.63±0.95	66.33±59.17	89.42±5.62
qHTT3.2- Hap1/*CHALK5/WX/ALK*	81.84±10.42**	62.76±14.67	22.63±0.95	66.33±59.17	89.42±5.62
qHTT4.1 -Hap1/*CHALK5/WX/ALK*	53.99±35.52**	58.53±12.64	23.07±0.91	61.8±50.41	89.42±5.62
qHTT4.2 -Hap1/*CHALK5/WX/ALK*	33.29±29.63*	50.47±18.2	22.89±3.11	33.66±19.46	89.34±4.53
qHTT3.1-X -Hap1/*CHALK5/WX/ALK*	81.84±10.42**	62.76±14.67	22.63±0.95	66.33±59.17	89.42±5.62
qHTT3.2- X-Hap1/*CHALK5/WX/ALK*	23.16±22.82	47.6±18.99	21.92±3.46	51.84±35.04	88.25±3.47
qHTT4 -X-Hap1/*CHALK5/WX/ALK*	30.08±25.57*	52.75±16.81	22.87±3.25	47.29±36.74	88.94±4.26
qHTT12 -X-Hap1/*CHALK5/WX/ALK*	42.99±16.75*	49.09±17.34	24.86±1.22	56.16±50.01	89.73±3.8
qHTT1- Hap2/*CHALK5/WX/ALK*	24.77±22.51	47.59±18.92	22.67±3.33	54.6±37.88	88.77±3.53
qHTT3.1- Hap2/*CHALK5/WX/ALK*	21.69±17.53	45.79±18.54	22.43±3.31	54.42±39.07	89.48±3.2
qHTT3.2- Hap2/*CHALK5/WX/ALK*	21.66±17.39	46.11±18.28	22.47±3.54	55.55±38.38	89.48±3.2
qHTT4.1- Hap2/*CHALK5/WX/ALK*	22.36±20.24	45.39±18.61	22.28±3.69	49.25±34.05	88.77±3.53
qHTT4.2- Hap2/*CHALK5/WX/ALK*	18.95±16.37	45.04±17.72	21.54±3.26	75.17±44.42	88.47±3.44
qHTT3.1 -X-Hap2/*CHALK5/WX/ALK*	20.62±17.32	46.15±17.76	22.34±3.52	54.5±37.96	89.48±3.2
qHTT3.2 -X-Hap2/*CHALK5/WX/ALK*	35.3±25.4	46.73±13.63	24.32±2.16	66.37±51.23	90.43±4.8
qHTT4 -X-Hap2/*CHALK5/WX/ALK*	13.55±12	34.03±12.9	20.71±3.31	86±36.75	88.55
qHTT12-X -Hap2/*CHALK5/WX/ALK*	22.29±23.55	47.05±18.01	21.97±3.45	55.07±37.42	88.08±4.11
qHTT1 -Hap1/*chalk5/wx/alk*	46.94±28.65	23.74±14.22	13.05±3.96	80.16±24.1	88.45
qHTT4.2 -Hap1/*chalk5/wx/alk*	50.48±25.87	24±16.92	14.01±4.85	72.6±26.62	89.9±1.99
qHTT3.2 -X-Hap1/*chalk5/wx/alk*	39.24±26	26.67±19.93	14.01±4.92	81.54±35.23	92.15±2.67
qHTT4 -X-Hap1/*chalk5/wx/alk*	42.53±25.4	30.29±19.53	13.39±4.58	79.64±31.7	90.86±2.62

qHTT1 -Hap2/*chalk5/wx/alk*	29.69±23.85	33.43±20.42	13.43±3.1	86.25±29.3	88.92±0.42
qHTT4.2 -Hap2/*chalk5/wx/alk*	20.4±14.7	37.41±19	12.92±3.75	89.5±32.29	94.7
qHTT3.2 -X-Hap2/*chalk5/wx/alk*	23.91±18.77	39.43±13.04	11.46±2.32	72.75±11.43	88.5
qHTT4 -X-Hap1/*chalk5/wx/alk*	10.05±6.46	23.78±13	16.2±0	74±12	-

Note: RHSR, HCD, HAC, HGC, HGT represent average relative seed setting rate under heat stress, chalkiness degree, amylose content, gel consistency, and gelatinization temperature under heat stress, respectively. * means significant difference between hap1 and hap2, ** means highly significant difference between hap1 and hap2.
