# Supplementary material for: Utilization of natural alleles for heat adaptability QTLs at the flowering stage in rice
Source: BMC Plant Biol. 2023 May 16;23:256. doi: 10.1186/s12870-023-04260-5 (PMC10186738; doi:10.1186/s12870-023-04260-5)
Supplement: Supplementary file 10 — Supplementary Material 10 [file 12870_2023_4260_MOESM10_ESM.pdf]

Table S6 Genotype of two varieties

**Name**	**118 you 726**	**Gui 726**
qHTT1	0	-
qHTT3.1	0	0
qHTT3.2	0	0
qHTT4.1	0	0
qHTT4.2	Heterozygosis	1
qHTT5	-	-
qHTT7.1	0	0
qHTT7.2	0	0
qHTT-X-3.1	1	1
qHTT-X-3.2		-
qHTT-X-3.3	0	0
qHTT-X-4	-	-
qHTT-X-5	0	0
qHTT-X-12	-	-
CHALK5	*chalk5*	*chalk5*
ALK	Heterozygosis	*alk*
WX	*Wx* -b	*Wx* -b
RHSR (%)	59.81%	69.47%
HCD	17.03	11.45
HAC (%)	10.93	14.42
HGC (mm)	116.00	131.00
HGT (℃)	88.55	93.25
