# Supplementary material for: Utilization of natural alleles for heat adaptability QTLs at the flowering stage in rice
Source: BMC Plant Biol. 2023 May 16;23:256. doi: 10.1186/s12870-023-04260-5 (PMC10186738; doi:10.1186/s12870-023-04260-5)
Supplement: Supplementary file 11 — Supplementary Material 11 [file 12870_2023_4260_MOESM11_ESM.pdf]

Table S7 Primers in the study

Primer	Sequence of primer (5' to 3')
UBQ F	ACCCTGGCTGACTACAACATC
UBQ R	AGTTGACAGCCCTAGGGTG
4-2880F	ACCGACGAGAGAAATGAAGC
4-2880R	CAGCTCCACTCCCAGTTTTG
4-2870F	TTCCGACCCAAAGAAAAATG
4-2870R	CCCCCACCTTTCACAGTAGA
4-2860F	GCGTGCAAATCCATTATGTG
4-2860R	TCGTTGCTTCCATGTCAGAG
4-2850F	GGAGATTTCACGCCTCTCAG
4-2850R	GCCTCTTCCTTTGCCTTCTT
4-2840F	CAAGCATGAAAACCTCGTCA
4-2840R	GCCTTTATGTCACGGTGGAT
4-2830F	GCGAGCAGATCAGACTACCC
4-2830R	CTGCACAAGAGAAGCACTCG
4-2820F	GTGGTGGAGAAAGAGCATCC
4-2820R	GCTCGAAATCTGCTCCAAAC
4-2810F	CACCAAGGGCAGTAGTCTCC
4-2810R	CTCAGCAGGTCCCTGAAGTC
4-2770F	ATGCAGAATTTGCACCCTTC
4-2770R	GAATTCCTCTCCGTGATCCA
4-2780F	ACAATCTTGACGGGGAGTTG
4-2780R	ATGACCTCCAGGTTCGTGAG
4-2790F	TACCTAGGCATCTCGGCTTC
4-2790R	GGGGAGAGGTGGAGGAAGTA
4-2800F	GATGGCGAGTTCGAGTTCAG
4-2800R	CTCGTCCTCTTCGACACGA
RWx-Fg	GAAGGTGACCAAGTTCATGCTTCATCAGGAAGAACATCTGCAAGG
RWx-Ft	GAAGGTCGGAGTCAACGGATTTCATCAGGAAGAACATCTGCAAGT
RWx-R	GGAAAAACGAGCAATGAAAGATGC
alk -Ftt	GAAGGTGACCAAGTTCATGCTTACAAGGAGAGCTGGAGGGGTT
alk -Fgc	GAAGGTCGGAGTCAACGGATTTACAAGGAGAGCTGGAGGGGGC
alk -R	CTGAGGTCCTGCGACATGC
Chalk5b -FT	GAAGGTCGGAGTCAACGGATTAGAGAGAAGTGCCAAGGATCTGT
Chalk5b -FC	GAAGGTGACCAAGTTCATGCTAGAGAGAAGTGCCAAGGATCTGC
Chalk5b -R1	TGCATCTAGCTACCTTCATTTCG
